# In Vivo Wound Healing and Immune Response Studies of Chitosan Cryogels With Invertebrate Model Organism *Galleria mellonella*

**DOI:** 10.1002/bip.70042

**Published:** 2025-07-30

**Authors:** Sema Ekici, Serhat Kaya, Gürkan Durucu

**Affiliations:** ^1^ Faculty of Sciences, Department of Chemistry, Hydrogel Research Laboratory Canakkale Onsekiz Mart University Canakkale Turkey; ^2^ Faculty of Sciences, Department of Biology, Insect Physiology Laboratory Canakkale Onsekiz Mart University Canakkale Turkey

**Keywords:** chitosan cryogel, full‐IPN cryogel, *G. mellonella*, hemostatic material, wound dressings

## Abstract

In the present study, it was aimed to prepare single and double network chitosan (Ch) cryogels cross‐linked with glutaraldehyde (G), which can be recommended for use as model wound dressings and hemostatic agents, and to reveal in vivo studies with *Galleria mellonella*. An in vivo study about Ch cryogels with these larvae was not declared in the literature, so our study is the first of its kind. *G. mellonella* was used to determine the effects of cryogels on immunity, oxidative stress, and wound healing. Cinnamic acid (CA) was loaded onto the cryogels, and the percent cumulative release data of CA were found to be in the range of 69%–80%. The results show that loading of CA onto [Ch‐3]_cry_ cryogels considerably improved immune responses; the [Ch‐3]_cry_‐CA group was the most successful in terms of immunological response, oxidative stress balance, and wound healing. In accordance with the 3R principles of ethical animal research, the use of *G. mellonella* in this study served as a scientifically relevant and ethically responsible alternative model to mammals for preliminary assessment of wound healing potential and innate immune activation. The porous structures, high mechanical strengths, and rapidly swelling‐deswelling abilities of [Ch‐2@Ch]_cry_ and [Ch‐3]_cry_ cryogels indicated that these may be suitable for biomedical applications. Analysis of SEM micrographs indicated that the morphology of dual network cryogels prepared in the form of interpenetrating polymeric networks (IPNs) was more regular and homodispersed with respect to single network cryogels. The compressive elasticity modulus (*E*) values of IPNs cryogels (0.160 N/mm) is approximately 4.6 times that of Ch cryogels with a single network (0.035 N/mm).

## Graphical abstract

– Comparison of chitosan cryogels: single networks versus full‐IPN structure.
– Cryogels displaying high swelling degrees more than 2800%, assigned to the interconnected macroporous structure like sponges.
– Cryogels with increased mechanical strength, more homogeneous and smaller pores in vivo wound healing and immune response studies of chitosan cryogels with *G. mellonella*.
– Enhanced the immune system, immunological and antioxidant defenses of *G. mellonella* via cryogels and their cinnamic acid‐loaded forms.

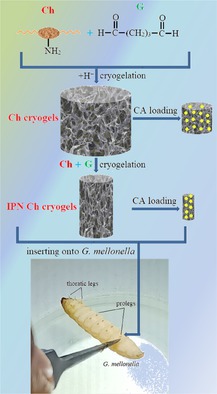

## 1 Introduction

Bleeding control is one of the most critical issues in civilian and military trauma centers worldwide [1]. Uncontrolled bleeding accounts for over 30% of traumatic deaths, with more than half occurring before reaching the emergency department [2]. In addition to this, every year, hundreds of people suffer from burns or other types of skin injury from fires, mishaps, and boiling water or oil, which frequently lead to disabilities from medical costs or even death. Currently, numerous materials are utilized and endorsed as haemostatic and burn dressings [3]. Tissue adhesives, albumin cross‐linked with glutaraldehyde, QuickClot containing zeolite, fibrin‐based bandages, and commercial or well‐known hemostatic materials, such as gelatin‐based hemostatic agents, have high hemostasis efficiency [4, 5, 6, 7].

Chitosan is unusual among medical materials because of its many useful uses, including similarity to extracellular matrix components, biocompatibility, biodegradability, non‐toxicity, and abundant properties [8, 9, 10]. Chitosan also exhibits antimicrobial activity arising from electrostatic interactions between positively charged amino groups of chitosan and negatively charged microbial cell membranes, resulting in increased cell wall permeability [11, 12]. By enhancing the functions of inflammatory cells such as polymorphonuclear leukocytes, macrophages, fibroblasts, or osteoblasts, chitosan supports wound organization and granulation, demonstrating high potential for wound healing [13, 14, 15]. Chitosan and chitosan‐derived wound dressings and haemostatic medical materials, which can be formulated as hydrogels, sponges, powders, membranes, fibres, microspheres, microneedles, aerogels, 3D printed dressings, and adhesives, garner significant interest from researchers owing to the advantageous properties of chitosan. The many morphologies of these materials each have their own set of benefits and drawbacks. The convenience of immediately applying haemostatic powders to wounds with irregular shapes is one example; nevertheless, after haemostasis, it is difficult to entirely remove the powder from the application area [16, 17, 18, 19]. On the other hand, chitosan hemostatic films, beads, membranes, and so forth, seem to have been replaced by chitosan cryogels because of their very weak mechanical strength; in other words, their brittleness. Chitosan cryogels as hemostatics and wound dressings appear to be the best candidates due to their superior wound‐healing properties. Chitosan‐based hemostatic cryogels ensure the aggregating of red blood cells, including polymorphonuclear leukocytes and macrophages, by stimulating platelets, resulting in effective rapid hemostasis [15, 20, 21, 22, 23, 24]. Chitosan cryogels could be prepared with a cryogelation technique that enables interconnected macroporous cavities, good mechanical performance, flexibility, and fast swelling‐deswelling behaviors owing to high porosity texture [23, 25, 26, 27]. Another good approach is the preparation of cryogels in the form of interpenetrating polymeric networks (IPNs) because IPNs could include characteristic properties of each polymeric component. The weak mechanical strength of this polycationic linear polysaccharide is an important shortcoming, so chitosan is rarely used alone, and attempts are made to compensate for this deficiency by adding other components. Different synthetic and natural/inorganic polymers such as polyethyleneimine [28], dextrin [29], poly(vinyl alcohol) [22, 30, 31, 32], Mxene [24], poly(hydroxyethy methacrylate) [33], sodium alginate [20], poly(acrylic acid) [21], and so forth, have been used for this aim. When comparing the pore sizes of different cryogels, it is observed that chitosan cryogels have larger pores than the others because of the large saccharide units on the main chain of chitosan. These saccharide rings of chitosan cause large pores to grow during cryogel formation, and the large spaces are very suitable for the placement of a second polymer chain [34]. In this way, the composite chitosan cryogels with increased mechanical strength and more functional and more homogeneous porosity are obtained.

This study intended to synthesize and characterize Ch cryogels in both single and full‐IPN forms, followed by an assessment of their in vivo cytotoxicity and antibacterial activity using *G. mellonella* as an invertebrate model. The use of invertebrate models such as *G. mellonella* allows for the initial screening of bioactivity, including wound healing potential, in a cost‐effective, ethically responsible, and biologically relevant manner. This model has been increasingly accepted for evaluating innate immune responses, tissue regeneration, and host‐pathogen interactions due to its physiological parallels with early vertebrate immune mechanisms and its ability to be maintained at mammalian body temperatures [35, 36, 37]. According to our research, no study on the interactions of Ch cryogels and CA‐loaded forms with *G. mellonella* has been presented in the literature. Therefore, our study will be the first research in this sense and will contribute to the literature.

The 3Rs principles (Replacement, Reduction, and Refinement) have driven the search for alternative model species to use in contemporary animal experiments [38]. According to the 3Rs, the greater wax moth (*G. mellonella*) has become a promising model among invertebrates in recent years [39, 40, 41]. *G. mellonella* serves as a viable alternative to conventional mammalian and non‐mammalian animal models, owing to its cost‐effectiveness, ease of manipulation, and significant biological characteristics for examining host–pathogen interactions [42]. In comparison to other invertebrate models, *G. mellonella* can tolerate temperatures up to 37°C, and its handling and experimental protocols are more straightforward, offering significant advantages, particularly in the study of human infections and the development of treatments targeting these pathogens [41].

In insects, immune responses are classified into two categories: humoral and cellular. According to recent research, both reactions depend upon circulating hemocytes. The total hemocyte count of *G. mellonella* is regarded as a significant indicator of immunity in this context [43, 44, 45]. Melanization, which is thought to be the most important humoral response [46], often accompanies the encapsulation response and plays a major role in isolating invaders that are too large to be phagocytosed from the hemocoel [47]. The removal of oxygen radicals produced during immune defense is essential for controlling oxidative stress and avoiding genotoxic harm. Antioxidant enzymes are essential to this crucial process, significantly influencing both pathogen invasion and wound healing [48]. We investigated the effects of cryogels on the immune responses of *G. mellonella* and the processes of wound healing and hemostasis, aiming to identify the component that enhances wound healing while elevating hemocyte count, melanization, and antioxidant enzyme activity.

## 2 Materials and Methods

### 2.1 Materials

Ch with high molecular weight (*M*_w_ ~ 600.000) (Fluka‐Steinheim, Switzerland) and glutaraldehyde (G) (Merck‐Schuchardt, Germany) were selected as polymer and crosslinker, respectively. Cinnamic acid (CA), *n*‐hexane, buffer components (Na_2_HPO_4_–KH_2_PO_4_), and products of Sigma‐Aldrich and Merck, respectively. Distilled water (DW) was used throughout the experiments. All chemicals were analytical grade and were studied as received.

### 2.2 Synthesis of [Ch]_cry_ and [Ch@Ch]_cry_ Cryogels

Deacetylated degree (DD%) of Ch determined via the potentiometric method was about 88%. The [Ch‐1]_cry_, [Ch‐2]_cry_, and [Ch‐3]_cry_ cryogels were synthesized as follows: The Ch solution was prepared by dissolving Ch in 2% acetic acid solution. Briefly, 8.0 g of Ch solution was used for all three cryogels, and the concentration of Ch solution was 0.55%, 1%, and 1.5% for [Ch‐1]_cry_, [Ch‐2]_cry_, and [Ch‐3]_cry_, respectively. Ch and G solutions (0.5 wt%) were mixed under continuous mixing (400 rpm) at room temperature. 275, 502, and 750 μL of G solutions were taken for three cryogels. After 1 h, the mixed solution was poured into injector moulds and left at −22°C for 24 h. Resulting cryogels were thawed at room temperature, and they were carefully removed from the moulds. Squeezed cryogels were thrown into DW for the washing procedure followed by drying in a lyophilizer at −55°C for 24 h.

To prepare [Ch‐1@Ch]_cry_ full IPN cryogels, dried [Ch‐1]_cry_ samples with long cylindrical shapes were waited in Ch solution (0.55%) until they were swollen. After this, swollen cryogels were left at −22°C for 24 h, again followed by the processes of thawing, washing, and lyophilization. [Ch‐2@Ch]_cry_ and [Ch‐3@Ch]_cry_ full IPN cryogels were prepared in the same way, except that the concentration of the Ch solutions in which they were swollen was different; that is, 1% and 1.5% of Ch solution were used for [Ch‐2@Ch]_cry_ and [Ch‐3@Ch]_cry_, respectively. All obtained cryogels with long cylindrical shapes were cut into pieces 5–8 mm in length following the washing and were stored in a refrigerator for further use.

### 2.3 Characterization of Ch Cryogels

#### 2.3.1 Scanning Electron Microscopy (SEM) Analysis

Cryogels dried in vacuum were dispersed on a Cu grid and coated with a 15 nm layer of Au‐Pd (80%–20%) alloy. SEM micrographics of cryogels were obtained with a LEOMODEL440 equipped with an Energy Dispersive System X‐Max Detector.

#### 2.3.2 Mechanic Tests

The compressive tests of the cryogels were realized with a universal testing machine (SHIMADZU AG‐XD 50 kN) at room temperature. Dry cryogels of 5–8 mm in length and 4–5 mm in diameter were selected for compressive analysis. The compression rate was fixed at 0.5 mm/min^−1^, and each analysis was repeated three times.

#### 2.3.3 Equilibrium Swelling Experiments

Because the cryogels have a spongy structure, they swell very quickly, and therefore swelling kinetics cannot be studied. The equilibrium swelling experiments were prepared as follows: DW was slowly dropped onto dried cryogel via a dropper until the cryogel was completely swollen and then the mass of the swollen cryogel was weighed again. The equilibrium swelling experiments were done with a non‐solvent (n‐hexane) for calculating porosity parameters. Swelling experiments with both DW and n‐hexane were repeated for four different cryogels to calculate standard deviations.

Pore volume per unit mass of dry cryogel, *V*_p_, was calculated following the equation:

(1) $$V_{p}=\frac{\left(m_{\mathrm{ns}}-m_{\mathrm{dry}}\right)}{m_{\mathrm{dry}}d_{1}}$$

In the equation, *m*_ns_ and *m*_dry_ are the weights of the cryogel in non‐solvent and dry, respectively, while *d*_1_ is the density of cyclohexane. Total porosity of dry cryogels (*P*); in other words, the volume of pores per unit volume of dry cryogel, was calculated with Equation (2);

(2) $$P=1-\frac{d_{0}}{d_{1}}$$

Here, *d*_0_ is the apparent density of the cryogel, while *d*_2_ is the density of the wall portions.

Porosity of the cryogel in swollen form is given by Equation (3);

(3) $$P_{s}=1-\frac{q_{v}}{1+\left(q_{w}-1\right)\left(d_{2}/d_{1}\right)}$$

The volume swelling ratio (*q*_v_), mass swelling ratio (*q*_w_), and the swelling degree (*S*_e_) of the cryogels were calculated using Equations (4–6):

(4) $$q_{v}={\left(D/D_{\mathrm{dry}}\right)}^{3}$$

(5) $$q_{w}=\left(m_{e}/m_{\mathrm{dry}}\right)$$

(6) $$S_{e}=\left(m_{e}-m_{\mathrm{dry}}\right)/m_{\mathrm{dry}}$$

where *m*_dry_ is the weight of dry cryogel, *m*_*e*_ is the weight of swollen cryogel at equilibrium time, *D* and *D*_dry_ are the radius of dry cryogel and swollen cryogel, respectively. The percent sol fraction (Sol Frac %) and the percent cryogel fraction (Cryo Frac %) were calculated following equations:

(7) $$\mathrm{Sol}\;\text{Frac}=\left[\left(m-m_{\mathrm{dry}}\right)/m_{\mathrm{dry}}\right]\times 100$$

(8) CryoFrac%=100−SolFrac%

where *m* represents the wet mass of cryogels after synthesis. The *V*_p_, *P*, *P*_s_, *S*_e_, and *Cryo Frac%* values calculated with standard deviations are given comparatively in Table 1.

**TABLE 1:** Porosity parameters of cryogels.

Cryogel	*P* cm^3^ porosity (cm^3^ dry cryogel)^−1^	*V*_p_ cm^3^ pore (g dry cryogel)^−1^	*P*_s_ cm^3^ porosity (cm^3^ wet cryogel)^−1^	*S*_e_ g solvent (g dry cryogel)^−1^	Cryo Frac %
[Ch‐1]_cry_	0.73 ± 0.05	36.95 ± 7.10	0.78 ± 0.01	58.80 ± 10.33	0.78 ± 2.55 × 10^−4^
[Ch‐1@Ch]_cry_	0.78 ± 0.03	29.19 ± 2.62	0.87 ± 0.02	49.70 ± 2.00	1.32 ± 7.06 × 10^−4^
[Ch‐2]_cry_	0.78 ± 0.02	36.18 ± 4.77	0.88 ± 0.02	55.39 ± 4.85	1.42 ± 3.06 × 10^−4^
[Ch‐2@Ch]_cry_	0.87 ± 0.01	25.31 ± 0.93	0.91 ± 0.02	39.30 ± 2.60	2.23 ± 1.00 × 10^−4^
[Ch‐3]_cry_	0.81 ± 0.06	22.86 ± 3.69	0.82 ± 0.02	34.18 ± 1.18	2.10 ± 3.00 × 10^−4^
[Ch‐3@Ch]_cry_	0.89 ± 0.01	22.71 ± 2.46	0.89 ± 0.01	28.69 ± 1.77	3.88 ± 2.80 × 10^−3^

#### 2.3.4 Drug Loading and Releasing Studies

Swollen cryogels with dry mass ranging from 150 to 200 mg were kept in 7 mL of 20 ppm CA solution, which was prepared in phosphate buffer (pH = 7.4), at +4°C for 24 h. After 24 h, absorptions of the CA solutions were read at *λ*_max_ = 272 nm using a UV–Visible spectrophotometer. A calibration graph was constructed by preparing the standard solutions, which have concentrations in the range of 1–10 ppm.

For kinetics release studies, CA‐loaded cryogels were put into vials containing 20 mL of PBS, and the vials were settled in a shaking water bath at 37°C. Three millilitres of the solution was taken from the medium at predetermined time intervals, and the absorptions were read at 272 nm. Three millilitres of fresh PBS was added to the release medium again, and the readings were continued until constant absorption values were reached.

The amount of adsorbed CA onto cryogels (Q) and the percent CA adsorption efficiency (AE%) were acquired using the following equations:

(9) $$Q=\frac{\left(C_{0}-C_{e}\right)\;V}{m}$$

(10) $$\mathrm{AE}\%=\frac{\left(C_{0}-C_{e}\right)\;}{C_{0}}\times 100$$

*Q* represents the amount of CA adsorbed onto cryogels. *C*_0_ and *C*_e_ refer to the initial and equilibrium concentrations of the CA solution, respectively. *V* is the volume of the CA solution, and m is the mass of dry cryogels.

The percent cumulative release *(CR%)* of CA from cryogels to solutions was calculated by:

(11) $$\mathrm{CR}\%=\frac{M_{t}\;}{M_{0}}\times 100$$

where *M*_*t*_ and *M*_0_ are the amount of drug released at time, *t* and in the cryogel, respectively.

### 2.4 In Vivo Wound Healing and Immune Response Studies

#### 2.4.1 *G. mellonella* Larvae Rearing

*G. mellonella*, in which achieved the last instar (0.2 ± 0.02 g), were selected from the continuing culture in the Insect Physiology Laboratory. The larvae were cultivated at a temperature of 30°C ± 1°C with a relative humidity of 65% ± 5% in constant darkness using artificial food [49] composed of wheat bran, naturally darkened honeycomb, water, honey, and plant‐derived glycerin. Three replicates were performed for each cryogel applied in all the experiments. Five samples were used per replicate (*n* = 15).

#### 2.4.2 Total Hemocyte Count Studies

To determine the total hemocyte count, cryogels with 0.1 mm diameter were inserted into the larvae's bodies through the anterior portion of their prolegs (Figure S1). After 24 pi, a small incision was punctured in the central region of the larvae, and 4 μL of hemolymph was collected. The hemolymph was transferred into microcentrifuge tubes containing 36 μL of anticoagulant solution (0.098 M NaOH, 0.186 M NaCl, 0.017 M Na_2_EDTA, and 0.041 M citric acid, pH 4.5) to ensure suspension. A 10 μL aliquot of the prepared suspension was placed in the counting area of a Neuber‐improved hemocytometer (Superior, Germany). The hemocyte concentration per mL was then determined using phase‐contrast microscopy (SopTop CX‐40, China) [38].

#### 2.4.3 Melanization Studies

The cryogels were cut into pieces about 0.1 mm in diameter and were placed inside the body from the anterior segment of the larvae's prolegs. Initial experiments demonstrated that 24 h was enough for assessing melanization; thus, this duration was chosen as the reference time. The melanization degrees of the cryogels were evaluated using a stereomicroscope (Leica EZ4, Germany). If melanization had not been observed on the cryogel or was present in a very little area, it was classified as none; if it was present in some regions, it was classified as partial; and if it was entirely melanized, it was classified as full (Figures S2 and 11).

#### 2.4.4 Antioxidant Activity

After cryogel pieces were inserted into the body cavity (24‐h), a small hole was created in the body of the larvae using a sterile needle, and 20 μL of the leaked hemolymph was collected. The hemolymph was then transferred into microcentrifuge tubes containing 180 μL of phosphate buffer with pH 7.4 and 0.01 mg of N‐phenylthiourea. The mixture was centrifuged at 10,000 rpm for 5 min (IKA, Germany). The resulting cell‐free supernatant was stored at −20°C for 10 days. The antioxidant activity was quantified using a microplate reader (Multiskan GO Microplate Spectrophotometer, Thermo Scientific, Finland) according to the methods of absorbance measurement achieved by Kaya [50].

#### 2.4.5 Wound Healing

Since there was no similar study in the literature, the experiment of wound healing was modified based on a study in the literature [51]. Besides, the injury model was based on a controlled puncture wound created on the larval body to mimic tissue damage, rather than employing a wound model; this approach was selected to investigate the local immune response and wound healing potential of the applied cryogels. In accordance with this, the larvae were first subjected to surface sterilization with absolute alcohol and then anesthetized by being kept on ice for 2 min. Then, a piece of cryogel was inserted into the hole opened in the body from the anterior segment of the prolegs, and the coagulation time of the hemolymph was determined. The onset time of coagulation was determined as the time of melanization occurred around the cryogel. To confirm coagulation, the cryogel was shifted through to the wound tissue, and it was checked whether hemolymph leakage occurred or not. The obtained data were recorded as minutes and seconds, and the results were evaluated with cluster analysis.

#### 2.4.6 Statistics

Cluster analysis was used to evaluate melanization status. A one‐way ANOVA analysis was performed for total hemocyte count, antioxidant activity except malondialdehyde (MDA), and wound healing time experiments, and the results were evaluated with the Tukey HSD test. MDA was evaluated with an independent t‐test due to its inhomogeneous distribution. The SPSS v22 (IBM, USA) program was used for statistical analysis. The results were visualized using a violin plot generated with R‐Studio.

## 3 Results

### 3.1 Designing of Single and Full‐IPN Ch Cryogels

**SCHEME 1:**
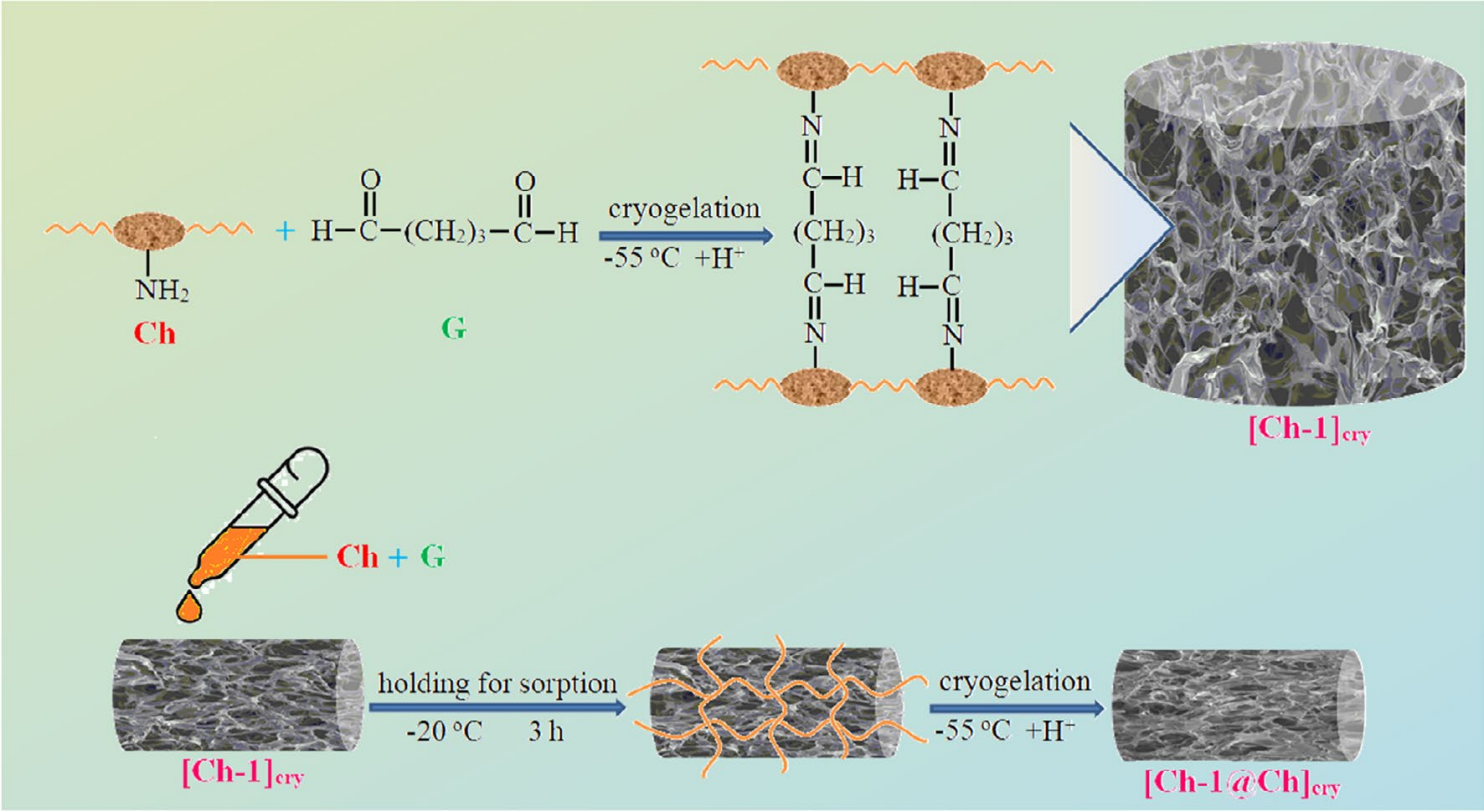
Synthesis procedure of Ch cryogels with single and full‐IPN structure.

Chitin, an amide derivative of glucose, is a cheap biopolymer that is found in shellfish such as crab, shrimp, and some species of fungi, and is the second most abundant after cellulose in nature. Ch is a linear polysaccharide containing *N*‐acetyl‐D‐glucosamine and D‐glucosamine monosaccharide side groups, obtained by deacetylation of the chitin biopolymer [9, 10]. So, it would not be wrong to say that Ch is a derivative biopolymer. G is a highly reactive crosslinker for Ch and some of the amine groups on Ch chains are cross‐linked with G in weak acidic medium [52]. The most characteristic feature of the cryogels is that they have huge pores allowing them to absorb solvents easily; in other words, they have a spongy structure. In the present study, these properties of cryogels were utilized for filling the big pores of the [Ch‐1]_cry_, [Ch‐2]_cry_, and [Ch‐3]_cry_ cryogels with Ch solution containing G, and this way, the [Ch‐1@Ch]_cry_, [Ch‐2@Ch]_cry_, and [Ch‐3@Ch]_cry_ full‐IPN cryogels were formed (Scheme 1).

**FIGURE 1:**
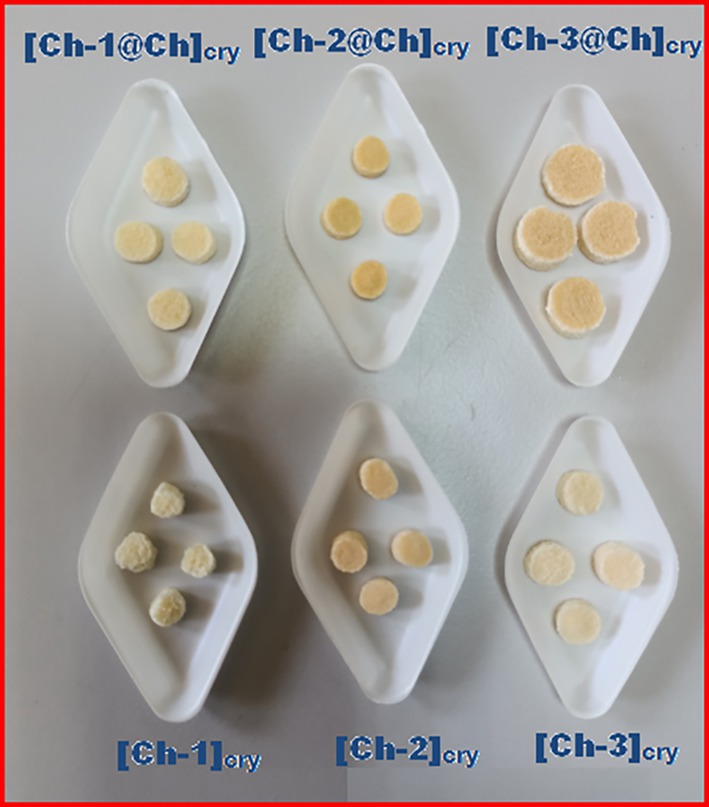
Photos of Ch cryogels after drying with lyophilizer. It is noteworthy that full‐IPN cryogels are larger and have more compact and smooth structure than single cryogels. As the amount of Ch in the cryogels increased, the color of the gels became more yellow due to iminization reaction between Ch and G in weakly acidic conditions.

Photographs and the stereomicroscope images of prepared cryogels taken after drying at −55°C in the lyophilizer are displayed in Figures 1 and S3, respectively. When [Ch‐1]_cry_, [Ch‐2]_cry_, and [Ch‐3]_cry_ cryogels are compared, it is understood that [Ch‐1]_cry_ has a looser texture and cannot maintain its shape integrity, even though they are all cryogelated in the same size mold. On the other hand, full‐IPN forms of these cryogels are more compact and can be handled more easily. As can be seen from Figure 1 and S3, [Ch‐3@Ch]_cry_ has a form with core‐shell morphology. The reason for this formation is that [Ch‐3]_cry_ sample cannot completely absorb the Ch solution poured on it during the preparation of [Ch‐3@Ch]_cry_ full‐IPN cryogel. If we compare the six cryogels, it would not be wrong to say that [Ch‐2@Ch]_cry_ and [Ch‐3]_cry_ samples have a regular texture and can be handled easily.

In Figure S4, the swelling and the compressive‐shape recovery behaviors of all cryogels are shown comparatively. Cryogels with a single network structure absorb and release water more rapidly than those with dual network structures due to the more compact structure of full‐IPNs. With even homogeneous porosity and good mechanical strength, full‐IPN cryogels are better. All cryogels preserved their physical integrity after repeated compression‐relaxation testing, with the exception of [Ch‐3@Ch]_cry_.

### 3.2 Porosity Studies

**FIGURE 2:**
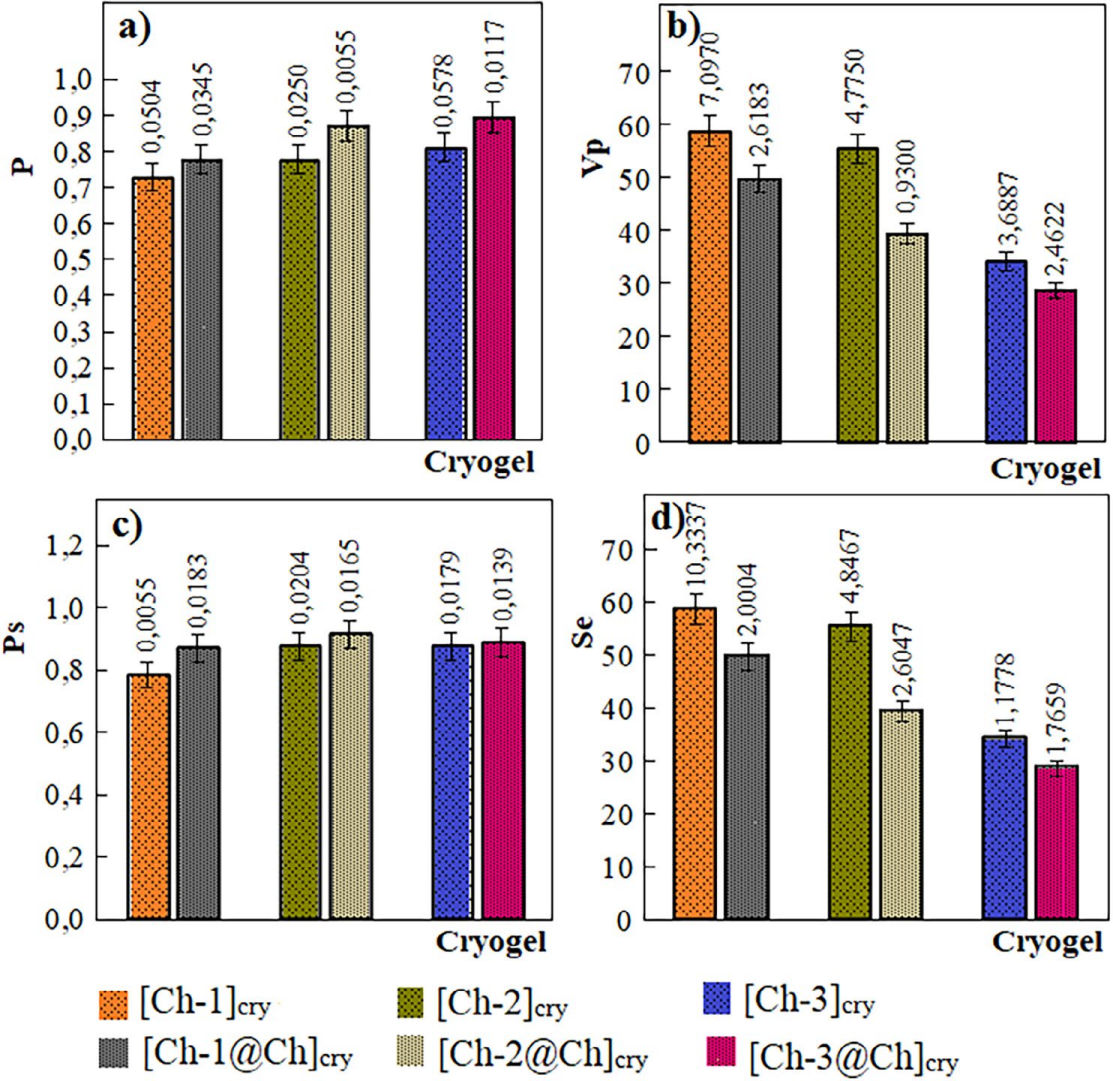
Comparative presentation of porosity parameters of cryogels. The legends given below graphs are valid for (a), (b), (c), and (d).

The results of simple measurements using distilled water and non‐solvent for the calculation of the basic porosity parameters such as *V*_p_, *P*, *P*_s_, *q*_v_, *q*_w_, and *S*_e_ of cryogels are given in Table 1. Besides, the graphs in Figure 2 were presented by constructing the data in Table 1.

Full‐IPN structures are synthesized in such a way that the first and second polymeric network structures are completely cross‐linked, and in this way more clamping of all polymer chains is achieved [53]. When the data of [Ch‐1]_cry_, [Ch‐2]_cry_, [Ch‐3]_cry_ cryogels, and their full IPN structures in Table 1 are compared, it is noteworthy that there are significant differences. For example, [Ch‐1]_cry_ has the lowest *P* (0.729 ± 0.050) and *P*_s_ (0.785 ± 0.005) values, while *P* (0.895 ± 0.012) and *P*_s_ (0.890 ± 0.014) values of [Ch‐3@Ch]_cry_ cryogel are the highest values. This is because it is the highest concentration of Ch in the [Ch‐3@Ch]_cry_ cryogel. As the volume of Ch in the cryogels increased, the volume of porosity per unit volume of both wet and dry cryogels increased. On the other hand, the pore volume per unit mass of the dry cryogel (*V*_p_ values) decreased owing to the raising crosslinks in the cryogel, confirming the characteristic feature for most cryogel materials [54, 55]. The similar comment is valid when the data of single and full‐IPN cryogels are evaluated too. For instance, the *P* and *P*_s_ values of the [Ch‐1]_cry_ cryogel are smaller than those of the [Ch‐1@Ch]_cry_ cryogel and the *P* and *P*_s_ values of the [Ch‐1@Ch]_cry_ are lower than those of the [Ch‐2@Ch]_cry_.

The degree of swelling (*S*_e_) decreases as the amount of crosslinking increases for both single and full‐IPN cryogels. This is clearly evident from the *S*_*e*_‐cryogel graph in Figure 2. The addition of a second Ch network to the single cryogel structure led to the reduction of the swelling capacity of the cryogels. The cryogels' swelling degrees gradually decreased from 58.797 ± 10.335 to 28.693 ± 1.766 due to the increased crosslinking density in the cryogels. All of the six cryogels displayed high swelling degrees of more than 2800%, assigned to the interconnected macroporous structure like sponges. Besides, the compressed cryogel could reach the swelling equilibrium between 1 and 10 s.

The order of Cryo Frac% values for single and dual series is as follows;

$${\left[\mathrm{Ch}-1\right]}_{\mathrm{cry}}<{\left[\mathrm{Ch}-2\right]}_{\mathrm{cry}}<{\left[\mathrm{Ch}-3\right]}_{\mathrm{cry}}$$

$${\left[\mathrm{Ch}-1@\mathrm{Ch}\right]}_{\mathrm{cry}}<{\left[\mathrm{Ch}-2@\mathrm{Ch}\right]}_{\mathrm{cry}}<{\left[\mathrm{Ch}-3@\mathrm{Ch}\right]}_{\mathrm{cry}}$$

In Table 1, the reason for the lower Cryo Frac% values is that the poor solubility of the Ch in acetic acid solution and, accordingly, the mass of Ch per unit volume in solution is very little in quantity. The majority of the Sol Frac consists of 2% acetic acid solution, which is used as a solvent, and the acetic acid solution has already been removed during washing and lyophilization. It is seen that the Cryo Frac% values increase as the single network changed to full‐IPN structure.

### 3.3 SEM Analysis

**FIGURE 3:**
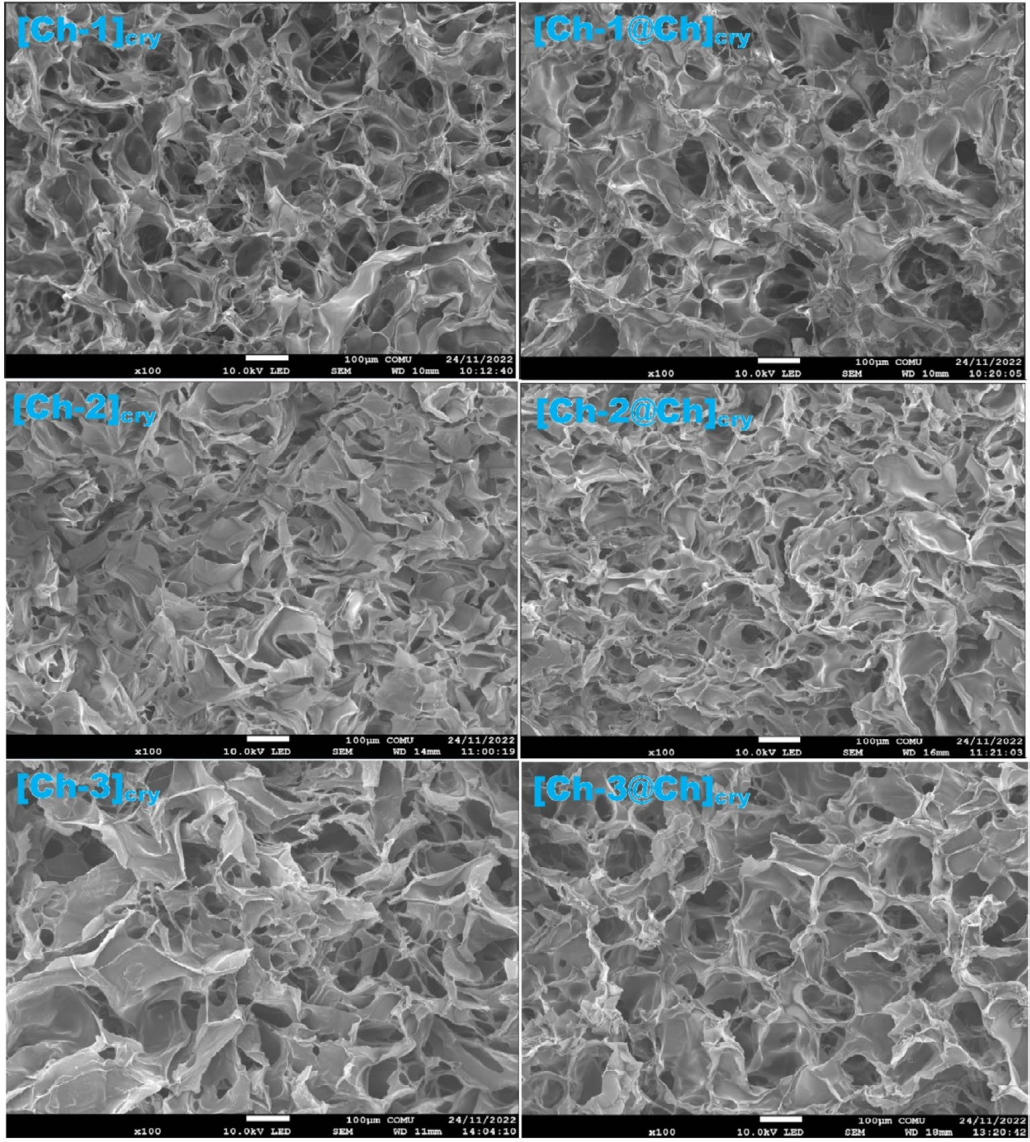
SEM images of cryogels taken at ×100 magnification. The bar indicates 100 μm.

The inner morphology of single and full‐IPN cryogels was also examined by SEM (Figure 3). All cryogels have a sponge‐like structure and contain interconnected super macroporous network structures [29]. We roughly estimated that the structures of [Ch‐1]_cry_, [Ch‐2]_cry_, and [Ch‐3]_cry_ cryogels were rearranged after the formation of the full‐IPN structure. The pore diameters and porosity of [Ch‐1]_cry_, [Ch‐2]_cry_, and [Ch‐3]_cry_ cryogels were also influenced by the addition of Ch. The single network structure transformed into a more closed form; in other words, the pores were closed or became small during the formation of [Ch‐1@Ch]_cry_, [Ch‐2@Ch]_cry_, and [Ch‐3@Ch]_cry_ full‐IPN cryogels. This formation also confirms the changes in the porosity parameter values given in Table 1. It is remarkable that the textures of [Ch‐2]_cry_ and [Ch‐2@Ch]_cry_ are different from the others, as can be explained in the comments of Figure 1 containing the photographs of cryogels. As expected for the full‐IPN structure, the higher the number of polymer chains the smaller the pore size [34].

### 3.4 Mechanical Analysis

**FIGURE 4:**
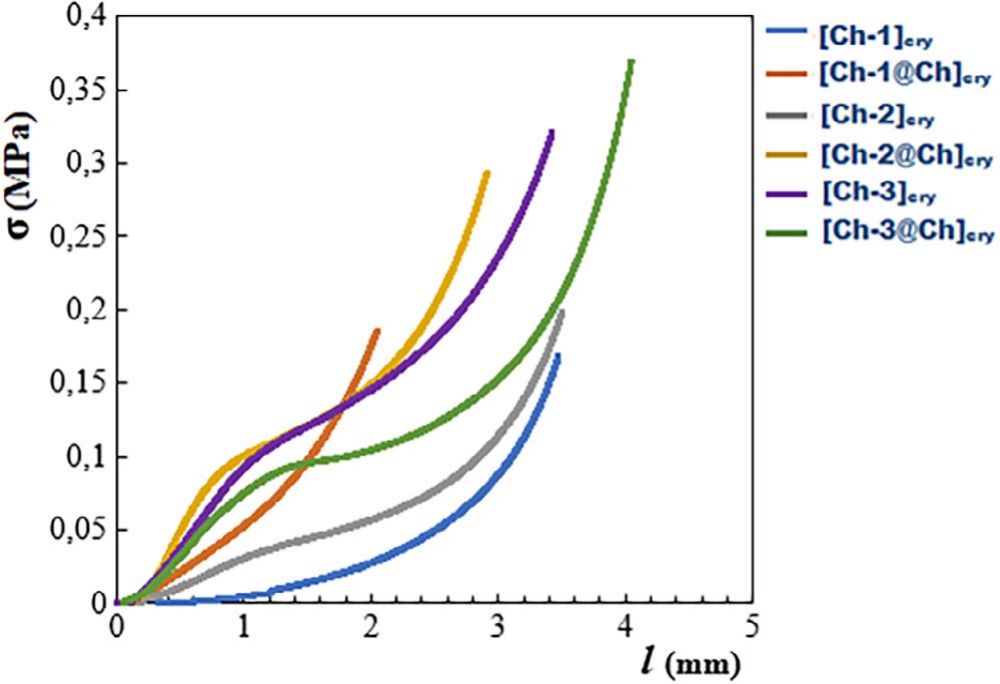
Curves of compressive strength (*σ*)–compressive strain (*l*) of cryogels.

It is important that the prepared cryogels do not disintegrate and maintain their integrity during the studies. With this aim, the compressive tests were applied to all cryogels and compressive strength (*σ*)–compressive strain (*l*, unit displacement) graphs were presented in Figure 4.

**FIGURE 5:**
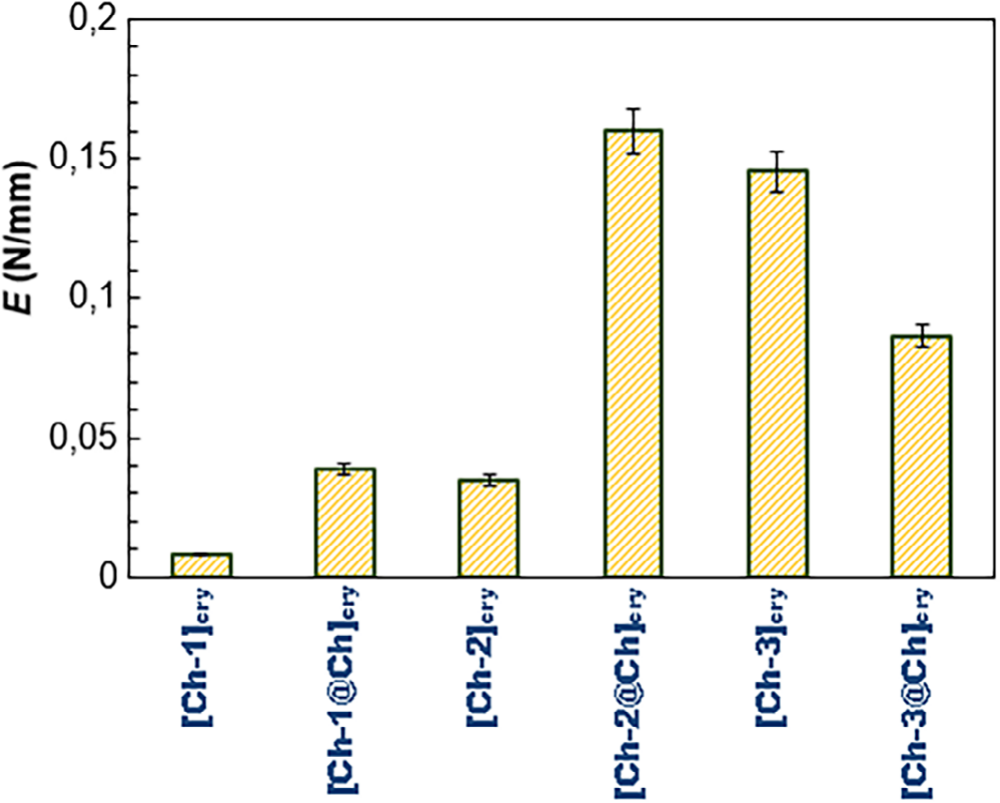
Comparison of *E* values of the cryogels.

As can be seen from the *σ*–*l* graphs in Figure 4, the most ductile cryogel is [Ch‐1]_cry_ cryogel, followed by [Ch‐2]_cry_ and [Ch‐1@Ch]_cry_ cryogels, respectively. [Ch‐2@Ch]_cry_, [Ch‐3]_cry_, and [Ch‐3@Ch]_cry_ cryogels are more stable than [Ch‐1]_cry_, [Ch‐2]_cry_, and [Ch‐1@Ch]_cry_ in terms of ductility. The compressive elasticity modulus (*E*) values calculated from the initial slopes of the elastic deformation region of the curves in Figure 4 are displayed in Figure 5.

The mechanical strengths of full‐IPN cryogels, except [Ch‐3@Ch]_cry_, are greater than those of single cryogels. For example, the *E* value of [Ch‐2@Ch]_cry_ cryogel (0.160 N/mm) is approximately 4.6 times that of [Ch‐2]_cry_ (0.035 N/mm). Preparation of Ch cryogels in full‐IPN structure had an increasing effect on their mechanical strength. On the other hand, the reason why [Ch‐3@Ch]_cry_ is mechanically weaker than [Ch‐3]_cry_ is that the dense Ch solution cannot completely diffuse into the pores of [Ch‐3]_cry_ during the preparation of full‐IPN structure. Furthermore, the cryogel with the highest mechanical strength is [Ch‐2@Ch]_cry_ followed by [Ch‐3]_cry_ cryogel. As can be understood from the cryogel photographs in Figure 1, the most regularly shaped samples are [Ch‐2@Ch]_cry_ and [Ch‐3]_cry_ cryogels, and they maintained their physical integrity during the repeated equilibrium swelling studies.

### 3.5 Drug Loading and Releasing Studies

CA was preferred as the drug active ingredient to be loaded onto the cryogels. CA is an organic compound found naturally in most plant sources and is usually obtained from cinnamon or frankincense. It has also been used for preparing toothpaste, mouthwash liquids, and chewing gum, cleaning materials, detergents, shampoos, perfumes, and cosmetics owing to its wide spectrum of biological activity, antioxidant, antimicrobial, and radical scavenging properties.

**FIGURE 6:**
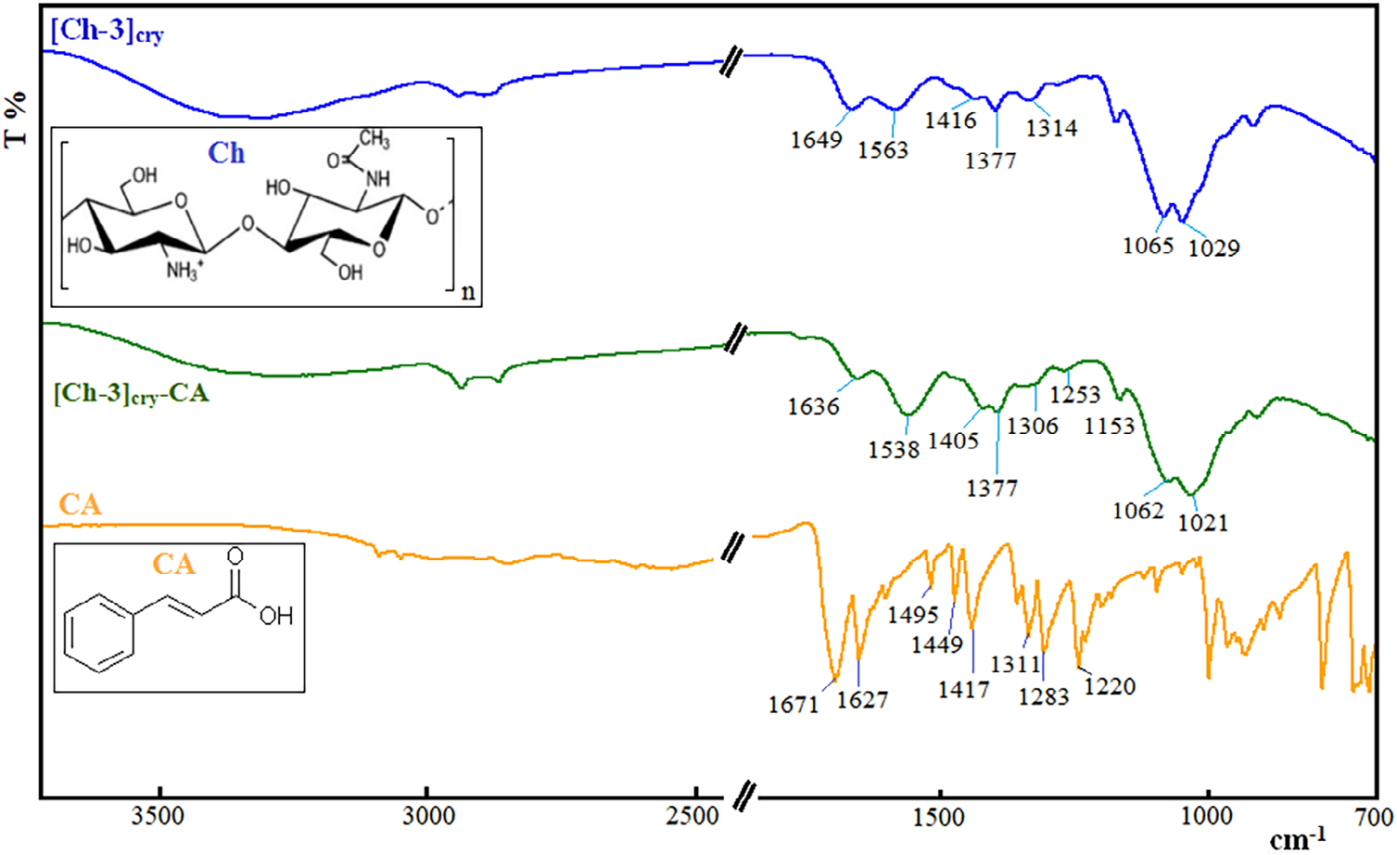
IR spectra of [Ch‐3]_cry_, [Ch‐3]_cry_‐CA, and CA.

To confirm the adsorption of CA molecules onto cryogels, infrared (IR) spectra of [Ch‐3]_cry_, [Ch‐3]_cry_‐CA, and CA were taken with an ATR‐connected FT‐IR spectrophotometer and are presented comparatively in Figure 6. In the spectrum of [Ch‐3]_cry_, the peak from 700 to 900 cm^−1^ is associated with C—H off‐plane bending vibrations, while the broad bands at 1029 and 1065 cm^−1^ show the stretching vibration of the —C—O group in the saccharide structure. Peaks at 1153 and 1314 cm^−1^ signal —C—O—C asymmetric stretching vibrations and —C—N stretching vibrations, respectively. The bending vibrations of —C—H groups are confirmed by the peaks located at 1377 and 1416 cm^−1^. Bands at 1649 and 1563 cm^−1^ are amide I (—C=O) and amide II (—N—H) associated with in‐plane stretching vibrations in the chitosan structure. The broad band between 3000 and 4000 cm^−1^ indicates the groups of —OH and —NH_2_ and the hydrogen bondings between these hydrophilic groups. In the spectrum of CA, the bands that are characteristic of the aromatic ring are located in the region of 1500–1600 cm^−1^. The band at 1671 cm^−1^ indicates the stretching vibration of the —C=O bond in the carboxylic acid group, while the band at 1627 cm^−1^ signals the stretching vibration of the —C=C— in the phenyl ring. The carboxylate anions give intense peaks between 1560 and 1610 cm^−1^. The bands at 1495 and 1449 cm^−1^ in the CA spectrum correspond to the stretching vibrations of the —C—H bonds. The bands in the 1420–1200 cm^−1^ range are the result of the coupling of —C—O stretching and —O—H in‐plane bending. The bands within the 700–1050 cm^−1^ fingerprint region are characteristic bands that are associated with the benzene ring. When the spectra of [Ch‐3]_cry_ and [Ch‐3]_cry_‐CA are compared, the most obvious difference is seen in the bands belonging to the amide groups of chitosan; in other words, the 1563 and 1649 cm^−1^ bands of [Ch‐3]_cry_ cryogel shift to the 1538 and 1636 cm^−1^ values in the spectrum of Ch‐CinA, respectively. In addition to this, the intensities and values of the bands for [Ch‐3]_cry_ in the range of 1200–1500 cm^−1^ also changed. These changes are due to the electrostatic interaction forces occurring between the protonated —NH_2_ groups of [Ch‐3]_cry_ and the —COO^−^ groups of CA. In addition to these, the 1416 cm^−1^ band in the Ch spectrum is accentuated in the [Ch‐3]_cry_‐CA spectrum as 1405 cm^−1^ owing to the presence of —CH groups in the CA structure. In light of these evaluations, it is understood that CA molecules are adsorbed onto the [Ch‐3]_cry_ cryogel as a qualitative assessment.

**FIGURE 7:**
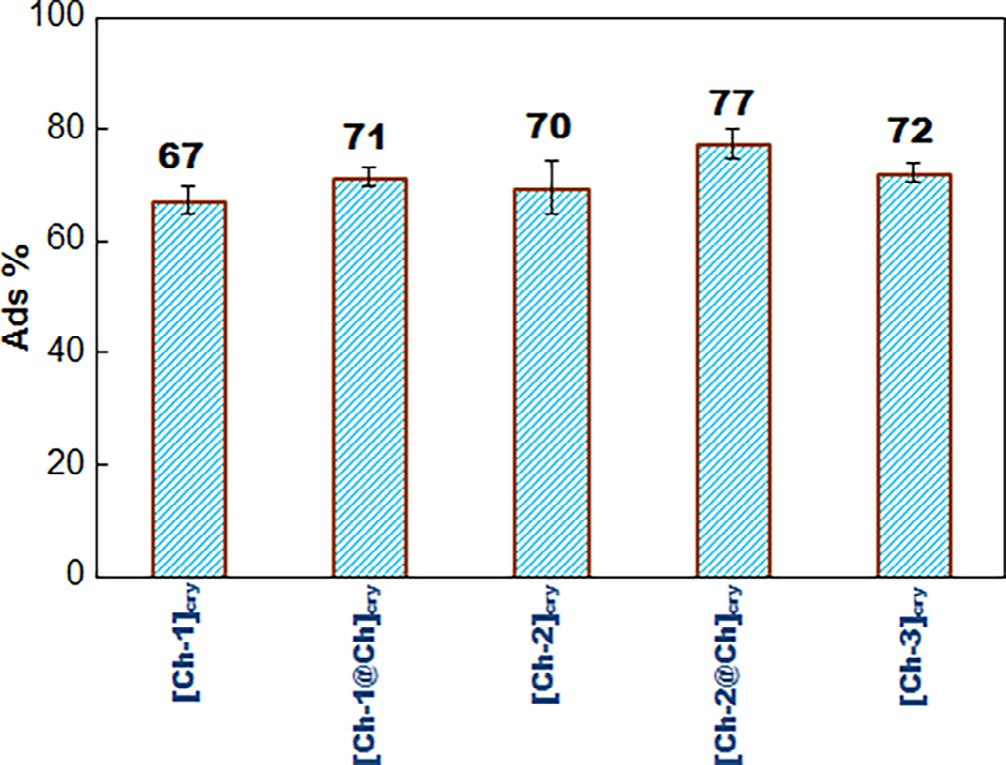
The plots of Ads% versus cryogels.

In order to quantitatively investigate the adsorption of CA molecules on to the cryogels, the equilibrium adsorption studies were carried out and the *Ads%‐Cryogel* graph was presented in Figure 7. The working with [Ch‐3@Ch]_cry_ cryogel was abandoned since there was disintegration/abrasion on its surfaces during both washing processes and CA loading studies. It is clear from Figure 7 that the cryogel with the highest CA adsorption is [Ch‐2@Ch]_cry_ cryogel owing to more functional groups in [Ch‐2@Ch]_cry_ compared to others.

**FIGURE 8:**
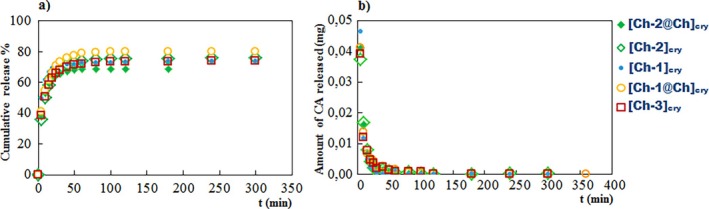
(a) The cumulative release graphs, (b) mg of released CA‐time graphs. Release medium: Simulated body fluid (pH 7.4).

Release of CA from the cryogels was examined as the percent cumulative release (CR%) and the amount of CA released (mg) parameters (Figure 8).

Release behavior of CA molecules from the cryogels obeys second‐order release kinetics, as can be concluded from Figure 8a. The percent cumulative release values of CA were found to be in the range of 69%–80%. The cryogel that releases the least CA molecules is [Ch‐2@Ch]_cry_ since its pore sizes are smaller and the porosity is higher than the other cryogels. It can be said that the release process for all samples is completed within the first 60 min. The largest amount of CA was released in the first 5 min from the [Ch‐1]_cry_ cryogel, which has the largest pores, and less mass of CA was released from the [Ch‐2@Ch]_cry_ with a compact structure (Figure 8b).

### 3.6 In Vivo Wound Healing and Immune Response Studies With *G. mellonella*

#### 3.6.1 The Survival Assay of *G. Mellonella*

**FIGURE 9:**
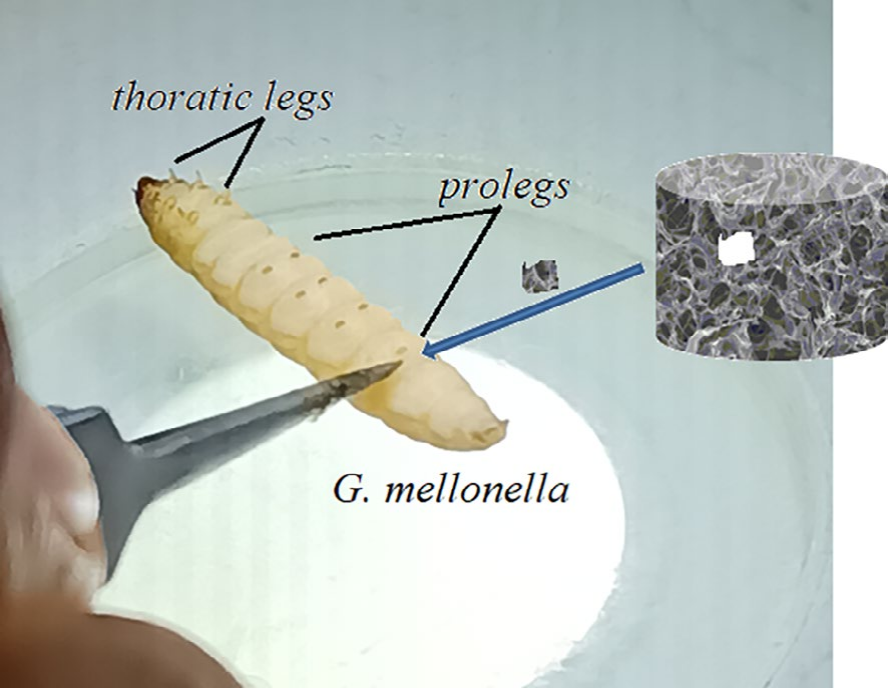
Inserting of cryogels on to the larva's leg.

There is no a research in the literature concerning the implantation of Ch cryogels onto *G. mellonella* larvae. So, our findings will be the first in this sense. The studies of wound healing and immune response were performed on [Ch‐3]_cry_ and [Ch‐2@Ch]_cry_ cryogels, which have higher mechanical strength and a more homogeneous structure, and their CA‐loaded samples (Figure 9). Firstly, in order to examine the biological effects and toxicity of cryogels and CA, a feeding assay was applied to the larvae. With this aim, *G. mellonella* larvae grown as described in the section of 2.4.1 were placed in petri dishes containing the [Ch‐3]_cry_, [Ch‐2@Ch]_cry_, [Ch‐3]_cry_‐CA, and [Ch‐2@Ch]_cry_‐CA cryogels (Figure S5). In the feeding tests, it was observed that the larvae ate all cryogels and consequently, no toxic effects were observed on the larvae.

#### 3.6.2 Total Hemocyte Count (THC) Assay

**FIGURE 10:**
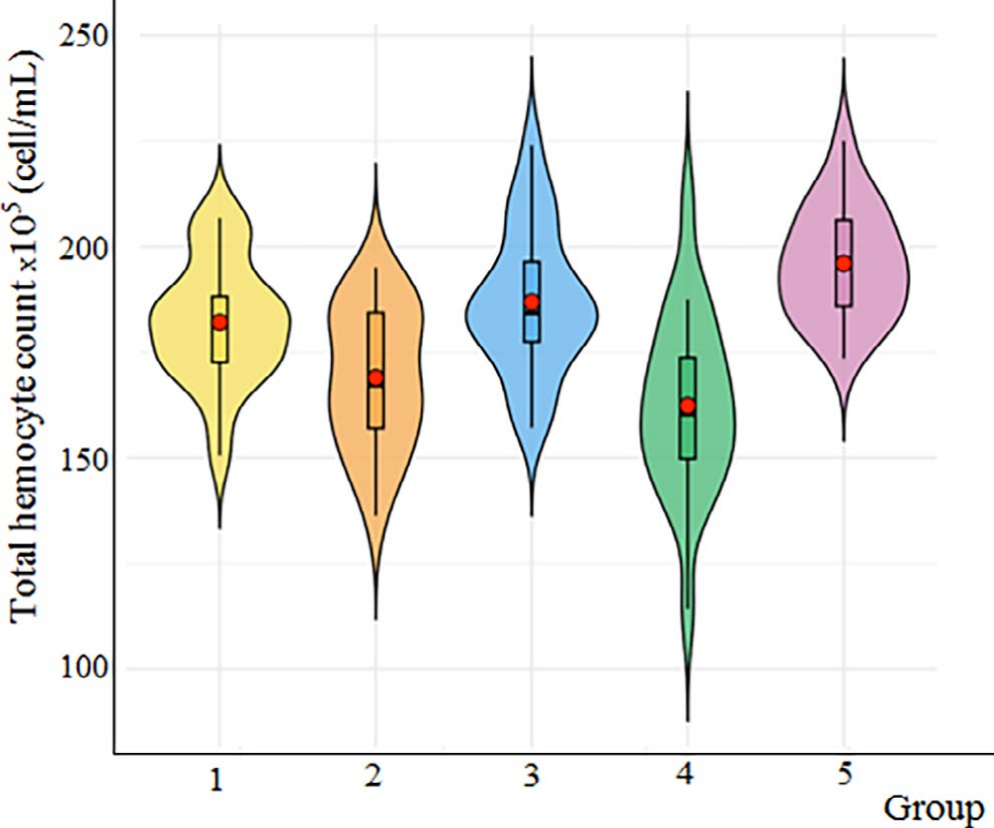
The effect of cryogel and CA on total hemocyte count of *G. mellonella*. The *x*‐axis indicates each of the experimental groups: 1, Untreated; 2, [Ch‐2@Ch]_cry_; 3, [Ch‐2@Ch]_cry_‐CA; 4, [Ch‐3]_cry_; 5, [Ch‐3]_cry_‐CA. Violin plot is a graphical representation of data distribution and density; the height of the box indicates data density at different value ranges, while the swollen regions represent distribution density. Variations in the height and distribution of data points across groups indicate differences in overall hemocyte count among the treatments. The central box in violin graph illustrates the interquartile range (IQR), the red dot represents the median, with the bold black line inside the box representing the average median. The whiskers extending above and below the central box denote the minimum and maximum values (excluding outliers).

Hemocytes serve as the primary defense against pathogens by secreting antimicrobial peptides, various humoral factors, and reactive oxygen intermediates, and they also eliminate them through phagocytosis and other cell‐mediated immune responses. The results demonstrating the effects of cryogel treatment on the total hemocyte count of *G. mellonella* are presented in Figure 10 via the violin graphs. It can be concluded that the [Ch‐3]_cry_‐CA cryogels exhibited a statistically significant difference, possessing a higher average total hemocyte count (196,87 × 10^5^ cell/mL) when compared to the other groups (*F* = 8.51, df1 = 4, df2 = 70; *p* < 0.001). The differences between other cryogels were determined to be insignificant, that is, the THC average data of other cryogels are 170.47 ± 5.31 × 10^5^ cell/mL, 162.47 ± 5.15 × 10^5^ cell/mL, 163.60 ± 5.51 × 10^5^ cell/mL and 172.60 ± 2.77 × 10^5^ cell/mL for [Ch‐2@Ch]_cry_‐CA, [Ch‐2@Ch]_cry_, [Ch‐3]_cry_, and the untreated control group, respectively.

The studies with invertebrate hemocytes reveal the critical role of hemocytes in immunity [56]. For example, the effects of zinc oxide nanoparticles (ZnO‐NPs) on *Bombyx mori* indicated a reduction in total and specific hemocyte counts, causing an enhancement in antioxidant activity at low doses and a decline at high doses [57]. In vitro studies in the literature show that CA induces apoptosis and can cause DNA damage [58]. Contrary to the literature knowledge, it was observed that CA caused an increase in hemocyte numbers in *G. mellonella*. Our in vivo studies indicate that CA could stimulate hemocyte proliferation at certain doses.

#### 3.6.3 Melanization Assay

**FIGURE 11:**
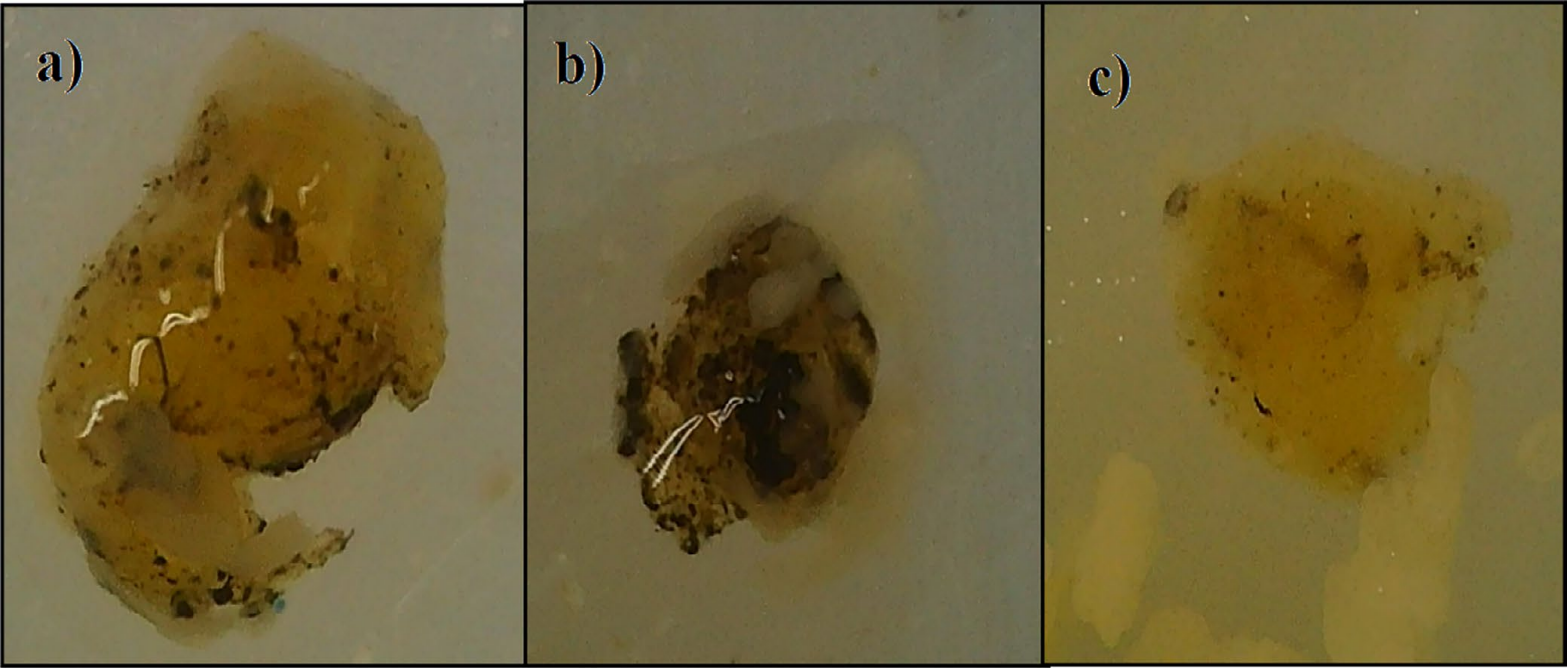
The melanization degrees of cryogels, (a) partial, (b) full, (c) none. The cream‐colored areas around the cryogel in (b) and (c) illustrate the larvae's fat bodies. Things resembling fabric are the reflections of the microscope's light.

The formation process of a melanized layer around invaders that are too large to be phagocytosed by hemocytes is known as melanization, resulting in isolation from the body. This process has been considered one of the most effective forms of humoral immunity in insects. Through the melanization process, the living being effectively protects its body against pathogens. Photographs of the cryogels, which were inserted into the larvae and removed after melanization, were shown in Figures 11 and S2. It was concluded that all cryogels had a melanization response; in other words, they were melanized.

**FIGURE 12:**
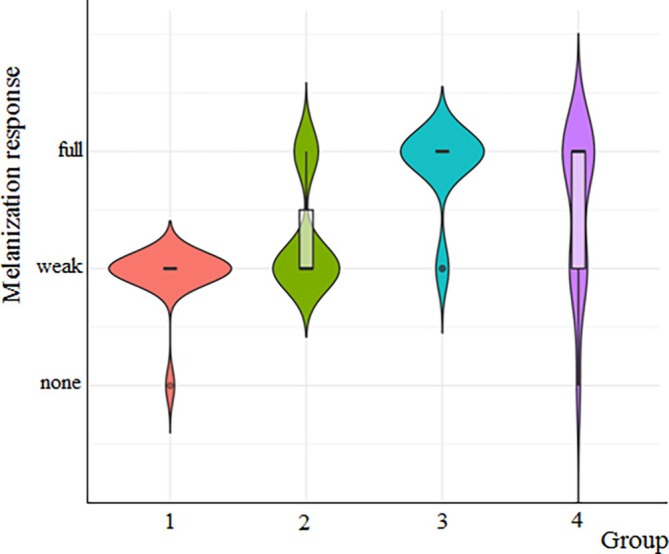
The effect of cryogel type and CA on melanization response of *G. mellonella*. The *x*‐axis indicates each of the experimental groups: 1, [Ch‐2@Ch]_cry_ (1 none/14 partial); 2, [Ch‐2@Ch]_cry_‐CA (4 partial/11 full); 3, [Ch‐3]_cry_ (2 partial/13 full); 4, [Ch‐3]_cry_‐CA (1 none/5 partial/9 full).

On the other hand, the melanization responses of the cryogels are shown comparatively in Figure 12. As can be seen from Figure 12, the [Ch‐3]_cry_ showed predominantly “strong” responses with a few “weak” responses. When [Ch‐3]_cry_ and [Ch‐2@Ch]_cry_ are compared, it can be said that the melanization level is higher for [Ch‐3]_cry_ cryogels in which the amount of Ch is larger and the pore sizes are smaller. Melanization enzymes (phenoloxidase) may have regarded the [Ch‐3]_cry_ cryogels as a support scaffold due to more compact structure of [Ch‐3]_cry_ cryogels.

The increasing of the melanization response is interpreted as a strengthening of immune responses [45, 59, 60]. When the literature was researched, no information was found regarding the effects of CA on the melanization of insects. Besides, in a study examining the relationship between the structure of CA derivatives and their tyrosinase inhibitory activity, it was emphasized that the changes of substituent groups and the C=C double bonds could affect both antioxidant and tyrosinase inhibitory activities, with specific compounds showing significant inhibition [61]. Tyrosine is the primary substrate for melanin synthesis during the melanization process [62]. Researching the melanization of *G. mellonella* with CA, we revealed that [Ch‐3]_cry_‐CA and [Ch‐2@Ch]_cry_‐CA cryogels had a higher melanization degree than the cryogels without CA. Furthermore, it can be concluded that CA has a positive effect on the melanization process, unless proven otherwise in the literature.

#### 3.6.4 Total Protein

**FIGURE 13:**
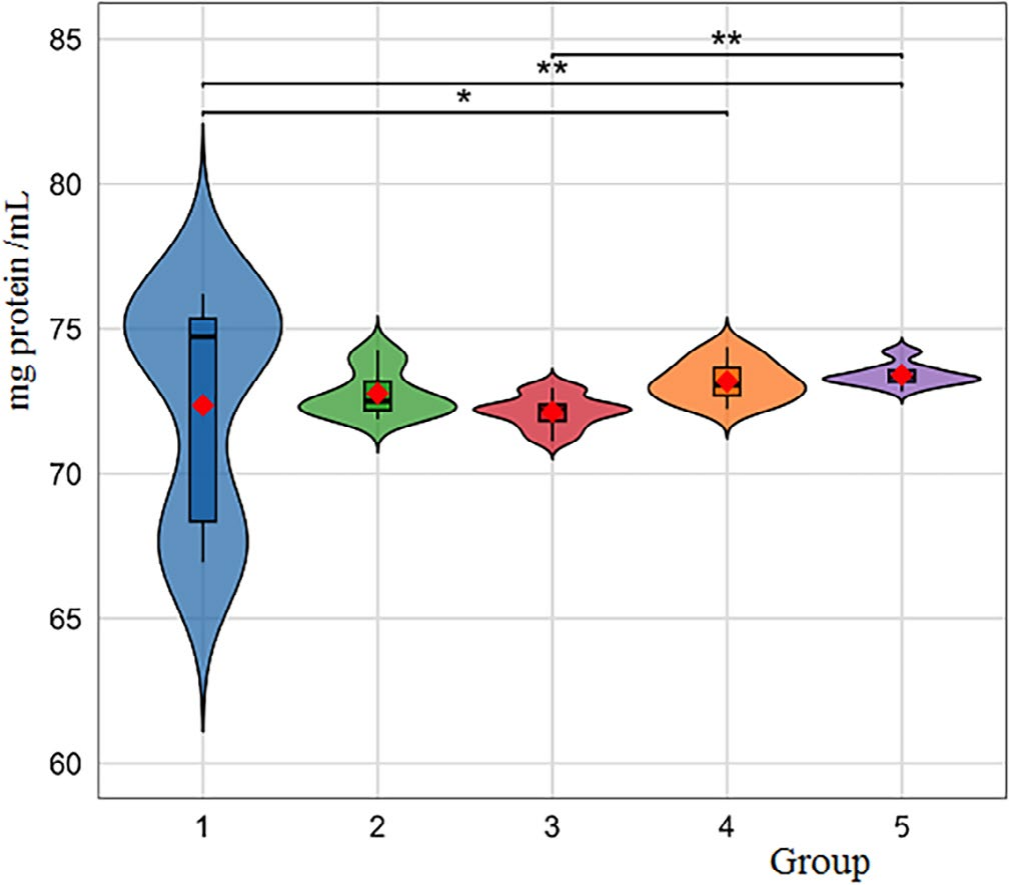
Violin plot representing the distribution of total hemolymph protein levels (mg/mL) in *G. mellonella* following different cryogel treatments. Each violin illustrates the probability density of the data at different values, with embedded boxplots indicating the interquartile range and median. Asterisks denote statistically significant differences (*p* < 0.05). 1; Untreated, 2; [Ch‐2@Ch]_cry_, 3; [Ch‐2@Ch]_cry_‐CA, 4; [Ch‐3]_cry_, 5; [Ch‐3]_cry_‐CA.

The effect of cryogel treatments on total hemolymph protein levels in *G. mellonella* is presented in Figure 13. The untreated control group (UNT) exhibited a mean protein concentration of 72.27 ± 3.92 mg/mL, with considerably higher variability compared to the cryogel‐treated groups (*p* < 0.05). Statistical analysis revealed no significant difference between the [Ch‐2@Ch]_cry_ (72.92 ± 0.20 mg/mL) and [Ch‐3]_cry_ (73.15 ± 0.17 mg/mL) groups (*p* = 0.743). However, the UNT group displayed significantly lower protein levels than both the [Ch‐3]_cry_ (*p* = 0.049) and [Ch‐3]_cry_‐CA (73.21 ± 0.15 mg/mL; *p* = 0.007) groups. Furthermore, a significant difference was found between the [Ch‐2@Ch]_cry_‐CA (72.55 ± 0.17 mg/mL) and [Ch‐3]_cry_‐CA groups (*p* = 0.004), with the latter exhibiting the highest protein levels among all groups. In contrast, the UNT group showed no significant difference from either the [Ch‐2@Ch]_cry_ (*p* = 0.812) or [Ch‐2@Ch]_cry_‐CA (*p* > 0.05) groups. Collectively, these findings indicate that all cryogel‐treated groups exhibited more stable and homogeneous protein levels compared to the untreated control, and that [Ch‐3]_cry_‐CA represents the most effective formulation for enhancing total hemolymph protein in *G. mellonella* (*p* = 0.004).

#### 3.6.5 Catalase (CAT) Assay

**FIGURE 14:**
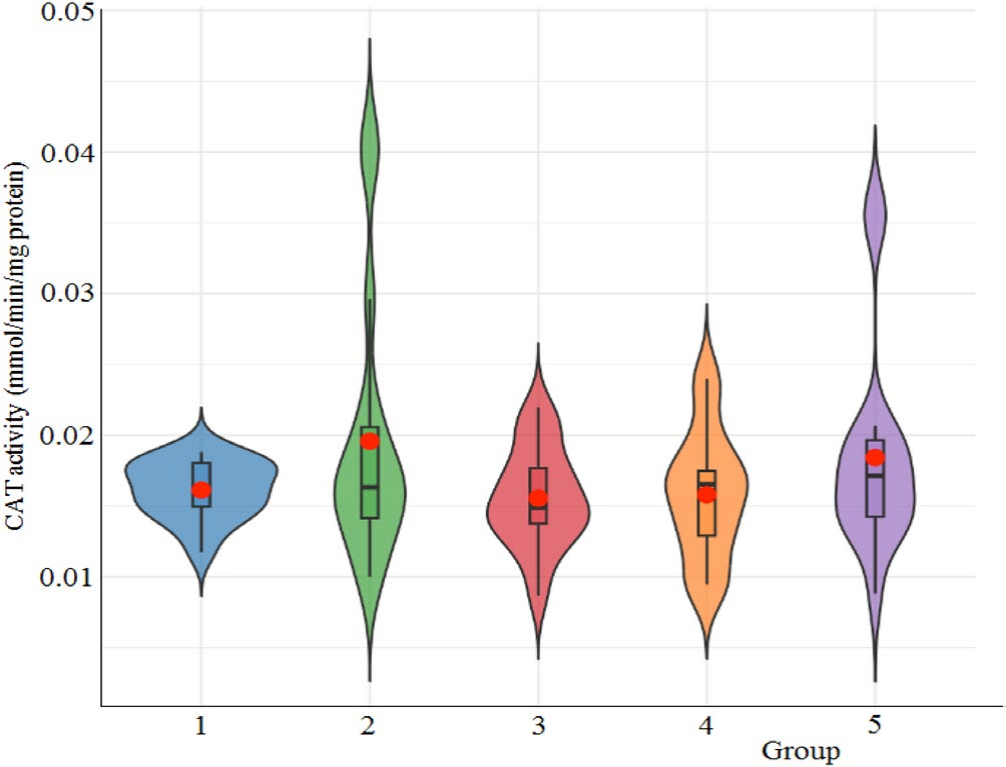
The effect of cryogel implantation on CAT activity for *G. mellonella* larval hemolymph. 1, Untreated; 2, [Ch‐2@Ch]_cry_; 3, [Ch‐2@Ch]_cry_‐CA; 4, [Ch‐3]_cry_; 5, [Ch‐3]_cry_‐CA.

The results showing the effect of cryogel on CAT activity in *G. mellonella* larval hemolymph are presented in Figure 14. The untreated control group (UNT) demonstrated a mean CAT activity of 0.0161 ± 0.0021 U/mg protein (mean ± SD), exhibiting the lowest variability among all experimental groups. Descriptive statistics revealed that CAT activity values ranged from 0.0152 to 0.0192 U/mg across the groups, with the [Ch‐2@Ch]_cry_ group showing the highest standard deviation (0.0096 U/mg), indicating substantial intra‐group variability. One‐way ANOVA indicated no statistically significant differences among the treatment groups (*F* = 1.305; df1 = 4, df2 = 75; *p* = 0.276), suggesting that the cryogel formulations did not produce measurable changes in CAT activity under the given experimental conditions. This finding was further supported by Welch's ANOVA for unequal variances (*F* = 1.21; df1 = 4, df2 = 31.7; *p* = 0.326) and the non‐parametric Kruskal–Wallis test (*χ* ^2^ = 5.63; df = 4; *p* = 0.228). Post hoc analysis confirmed the absence of significant pairwise differences (all *p* > 0.05).

Notably, while the [Ch‐2@Ch]_cry_ group showed the highest mean activity (0.0192 U/mg), this increase was not statistically significant compared to controls (*p* = 0.682). The UNT group exhibited the most consistent distribution (95% CI: 0.0151–0.0171), whereas [Ch‐3]_cry_‐CA (0.0184 ± 0.0079 U/mg) and [Ch‐2@Ch]_cry_‐CA (0.0152 ± 0.0036 U/mg) displayed differential variability patterns despite similar mean activities. In conclusion, while cryogel treatments did not significantly modulate CAT activity in *G. mellonella*, group‐specific differences in response variability may reflect subtle influences of cryogel composition on the insect's antioxidant defense system. Further studies with larger sample sizes and refined conditions could help clarify these observations. In reaction to oxidative stress, organisms can utilize superoxide dismutase (SOD) and catalase (CAT) to eliminate reactive oxygen species (ROS) and so safeguard cellular homeostasis [63].

#### 3.6.6 Superoxide Dismutase (SOD) Assay

**FIGURE 15:**
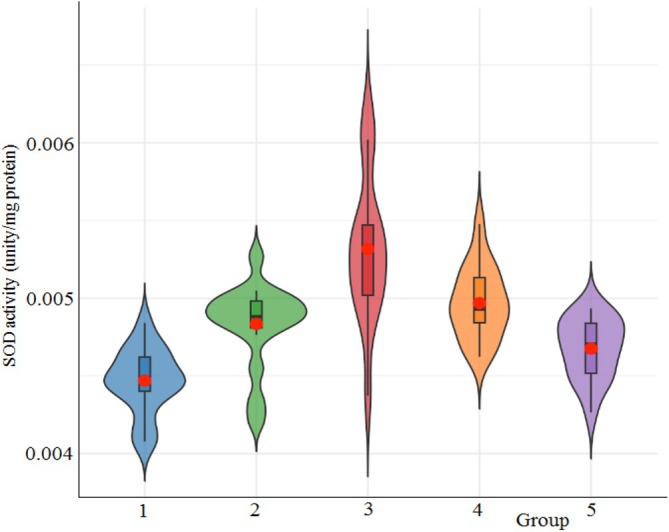
Impact of cryogel on SOD activity for *G. mellonella* larval hemolymph. 1, Untreated; 2, [Ch‐2@Ch]_cry_; 3, [Ch‐2@Ch]_cry_‐CA; 4, [Ch‐3]_cry_; 5, [Ch‐3]_cry_‐CA.

The activity of SOD is crucial for the removal of oxygen radicals from hemolymph and their change into H_2_O_2_, thus safeguarding cells from genotoxic damage. The effect of cryogel on SOD activity in larval hemolymph of *G. mellonella* is depicted in Figure 15. Among the experimental groups, [Ch‐2@Ch]_cry_‐CA exhibited the highest mean SOD activity (0.0053 ± 0.0005 U/mg protein), while the untreated control group demonstrated the lowest (0.0045 ± 0.0002 U/mg protein). Descriptive analysis showed that the SOD activity across groups ranged narrowly from 0.0045 to 0.0053 U/mg protein, with [Ch‐3]_cry_‐CA displaying the lowest variability (standard deviation = 0.0002), and [Ch‐2@Ch]_cry_‐CA showing the widest dispersion. One‐way ANOVA revealed a statistically significant difference among the groups (*F* = 15.42, df1 = 4, df2 = 75, *p* < 0.001), indicating that cryogel composition had a notable effect on SOD activity. Post hoc Tukey HSD analysis demonstrated that [Ch‐2@Ch]_cry_‐CA had significantly higher SOD activity compared to the untreated control (*p* < 0.001), [Ch‐3]_cry_ (*p* = 0.012), and [Ch‐3]_cry_‐CA (*p* = 0.001). Additionally, the [Ch‐3]_cry_ group also showed significantly elevated activity compared to the untreated group (*p* = 0.003). These findings highlight the potent antioxidant effect of [Ch‐2@Ch]_cry_‐CA, suggesting that this formulation may offer superior protection against oxidative stress in *G. mellonella* by enhancing SOD‐mediated radical scavenging. The consistent elevation in SOD activity observed in [Ch‐2@Ch]_cry_‐CA underscores its potential utility in biomaterial applications aimed at immunological and oxidative stress modulation.

The research conducted with STZ‐induced diabetic mice found that lipid peroxidation and ROS levels increased while CAT‐SOD activity decreased; nevertheless, CA administration improved the CAT‐SOD activity and reduced lipid peroxidation and ROS levels [64]. A study evaluating the effects of cinnamic aldehyde and CA derived from *Cinnamomum cassia* on isoproterenol‐induced myocardial ischemia in rats demonstrated that both compounds enhanced SOD activity and reduced MDA levels in cardiac tissue [65]. Our results showed that SOD activity significantly increased in CA‐loaded cryogels, particularly in [Ch‐2@Ch]_cry_‐CA. In addition, [Ch‐3]_cry_ also demonstrated elevated SOD activity, suggesting that both cryogel structure and CA loading contribute to enhanced antioxidant capacity.

#### 3.6.7 Malondialdehyde (MDA) Assay

**FIGURE 16:**
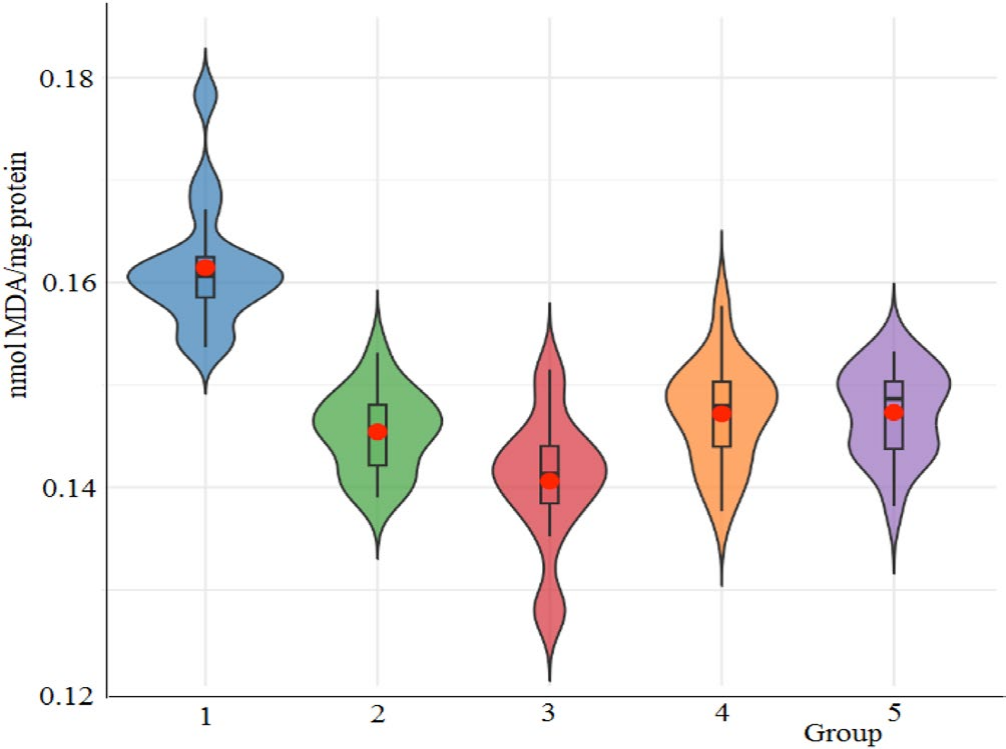
Comparison of the cryogels effects on MDA levels of *G. mellonella* hemolymph. 1, [Ch‐2@Ch]_cry_; 2, [Ch‐2@Ch]_cry_‐CA; 3, [Ch‐3]_cry_; 4, [Ch‐3]_cry_‐CA. 1, Untreated; 2, [Ch‐2@Ch]_cry_; 3, [Ch‐2@Ch]_cry_‐CA; 4, [Ch‐3]_cry_; 5, [Ch‐3]_cry_‐CA.

In order to investigate the lipid peroxidation effects of the cryogels, malondialdehyde (MDA) levels in larval hemolymph were evaluated using spectrophotometric analysis (Figure 16). The highest MDA concentration was observed in the untreated group (0.1609 ± 0.0068 mmol/mg protein), whereas the lowest value was detected in the [Ch‐2@Ch]_cry_‐CA group (0.1408 ± 0.0071 mmol/mg protein). Descriptive statistics revealed that [Ch‐2@Ch]_cry_‐CA not only exhibited the lowest MDA levels but also differed significantly from several other groups, as shown by one‐way ANOVA results (*F* = 22.87, df1 = 4, df2 = 75, *p* < 0.001). Tukey HSD post hoc analysis confirmed that the untreated group had significantly higher MDA levels than all other cryogel‐treated groups (*p* < 0.001 for each comparison). Additionally, a significant difference was observed between [Ch‐3]_cry_ and [Ch‐2@Ch]_cry_‐CA (*p* = 0.021), suggesting enhanced lipid peroxidation suppression with CA loading onto the interpenetrating network structure. While the MDA levels in [Ch‐3]_cry_ (0.1469 ± 0.0056 mmol/mg protein), [Ch‐3]_cry_‐CA (0.1469 ± 0.0048 mmol/mg protein), and [Ch‐2@Ch]_cry_ (0.1447 ± 0.0042 mmol/mg protein) did not differ significantly among themselves (*p* > 0.05), [Ch‐2@Ch]_cry_‐CA consistently outperformed all other formulations in reducing MDA levels, supporting its superior antioxidative potential.

These findings collectively demonstrate that the incorporation of CA into the [Ch‐2@Ch]_cry_ structure notably enhances its ability to counteract lipid peroxidation. This effect is likely mediated by CA's free radical scavenging capacity and synergistically supported by the structural properties of the cryogel network. Therefore, [Ch‐2@Ch]_cry_‐CA emerges as a promising candidate for applications requiring oxidative stress mitigation. The CA nanoparticles reduce the severity of acute pancreatitis by regulating redox pathways and MDA levels too [66].

#### 3.6.8 Wound Healing

**FIGURE 17:**
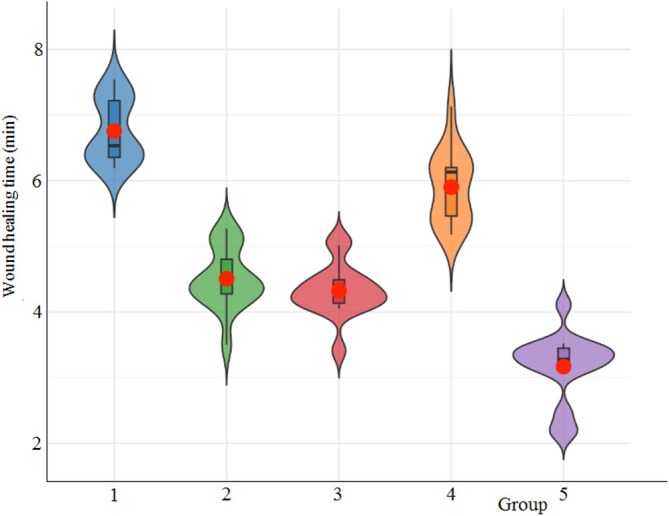
Effect of cryogel application on wound healing rate in *G. mellonella*. 1, Untreated; 2, [Ch‐2@Ch]_cry_; 3, [Ch‐2@Ch]_cry_‐CA; 4, [Ch‐3]_cry_; 5, [Ch‐3]_cry_‐CA.

To examine the wound healing characteristics of [Ch‐2@Ch]_cry_ and [Ch‐3]_cry_, along with their CA‐loaded variants, [Ch‐2@Ch]_cry_‐CA and [Ch‐3]_cry_‐CA, the cryogels were implanted at the wound site on the anterior segment of the larvae's prolegs, and the hemolymph clotting time was documented. Figure 17 highlights that statistically significant differences have been identified across all experimental groups, including the untreated control (UNT) (*F* = 79.00; df1 = 4, df2 = 75; *p* < 0.001). The [Ch‐3]_cry_‐CA group exhibited the lowest clotting time (3 min 17 s), significantly outperforming all other groups (*p* < 0.001). The [Ch‐2@Ch]_cry_‐CA and [Ch‐2@Ch]_cry_ groups had moderate healing durations of 4 min 33 s and 4 min 51 s, respectively, both considerably less than the untreated control at 6 min 28 s (*p* < 0.001). The [Ch‐3]_cry_ group exhibited a protracted healing response (5 min 59 s), which was significantly separate from both [Ch‐3]_cry_‐CA and [Ch‐2@Ch]‐based cryogels, yet not significantly different from the UNT group (*p* > 0.05), suggesting that the unmodified [Ch‐3]_cry_ formulation did not enhance wound healing. The results indicate that CA inclusion markedly improves clotting efficiency, especially in the [Ch‐3]_cry_ matrix, presumably owing to its anti‐inflammatory characteristics and its capacity to regulate haemocyte dynamics. All CA‐loaded cryogels markedly decreased clotting time relative to the control, indicating their potential to facilitate rapid wound closure in *G. mellonella*. Although clotting time serves as a valuable indicator of wound healing, further investigations, including tissue remodeling and cellular infiltration, are essential to comprehensively clarify the underlying biological mechanisms.

## 4 Discussion and Conclusion

Herein, we proposed to evaluate the properties of Ch cryogels prepared in single and full‐IPN structures and to compare the interactions of bare Ch and CA‐loaded cryogels with *G. mellonella*, which could serve as an alternative in vivo model owing to its ease of growing and handling, low cost, and simpler experimental protocols. An increase in the Ch concentration of the [Ch‐1]_cry_, [Ch‐2]_cry_, and [Ch‐3]_cry_ cryogels led to a decrease in the swelling degree. When the cryogels with single and full‐IPN structures were compared among themselves, it was observed that the degree of swelling (*S*_e_) and pore sizes (*V*_p_) decreased as the amount of Ch in the cryogel increased. SEM analyzes revealed that full‐IPN cryogels had a more homogeneous and tight structure as expected. In addition to these, the preparation of Ch cryogels in full‐IPN structure had an increasing effect on their mechanical strength. For example, the *E* value of [Ch‐2@Ch]_cry_ cryogel (0.160 N/mm) is approximately 4.6 times that of [Ch‐2]_cry_ (0.035 N/mm). In accordance with the 3R principles of ethical animal research, the use of *G. mellonella* in this study served as a scientifically relevant and ethically responsible alternative model to mammals for preliminary assessment of wound healing potential and innate immune activation. This model not only ensured ethical compliance but also yielded meaningful biological insights. Using this model, experimental results demonstrated that the [Ch‐3]_cry_‐CA group was the most successful in terms of immune response, oxidative stress balance, and wound healing. Using *G. mellonella* as a model, clotting time analysis demonstrated formulation‐dependent differences in wound closure efficiency. The [Ch‐3]_cry_‐CA group exhibited the shortest clotting time (3 min 17 s), significantly outperforming all other groups including the untreated control. Besides, the loading of CA onto the [Ch‐3]_cry_ further strengthened the immune responses. However, a closer look at the melanization data reveals that [Ch‐3]_cry_ without CA loading showed the highest proportion of strong melanization responses (13/15), exceeding that of both CA‐loaded groups ([Ch‐2@Ch]_cry_‐CA with 11/15 and [Ch‐3]_cry_‐CA with 9/15). Notably, [Ch‐3]_cry_, despite inducing strong melanization, did not significantly differ from the untreated group in clotting time (*p* > 0.05), suggesting that melanization alone may not be predictive of wound closure efficiency. This indicates that the effect of CA on melanization is not uniformly enhancing across all cryogel formulations. In fact, CA loading enhanced melanization in the [Ch‐2@Ch]_cry_ formulation but appeared to reduce it in the [Ch‐3]_cry_ formulation. These findings suggest that CA's influence on melanization is modulated by the structural and compositional characteristics of the cryogel, warranting further mechanistic studies. Notably, [Ch‐3]_cry_‐CA demonstrated a significantly higher total hemocyte count not only compared to [Ch‐3]_cry_, but also when compared to all other groups, underscoring its potent immunostimulatory effect. In addition to these, the SOD activity of [Ch‐2@Ch]_cry_‐CA was significantly higher than those of [Ch‐3]_cry_, [Ch‐3]_cry_‐CA, and the untreated control, highlighting the enhanced antioxidant activity achieved by CA loading in the full IPN cryogel structure. While [Ch‐3]_cry_‐CA showed strong immune and healing performance, it is important to emphasize that [Ch‐2@Ch]_cry_‐CA exhibited the most robust antioxidant response among all groups, as evidenced by both elevated SOD activity and lowest MDA levels. It is noteworthy that CAT activity did not significantly differ among the treatment groups, suggesting that the antioxidant effect of the cryogels was primarily reflected in SOD activity and lipid peroxidation reduction, rather than in catalase‐mediated mechanisms. Although not statistically significant, the observed variations in CAT activity distribution among the groups may suggest underlying biological responses to different cryogel formulations, particularly in terms of redox homeostasis. Furthermore, the [Ch‐3]_cry_‐CA group showed the highest total hemolymph protein concentration, indicating enhanced systemic protein expression, which may be associated with improved immune readiness or metabolic activation in response to cryogel treatment. Interestingly, the total protein levels in [Ch‐2@Ch]_cry_ and [Ch‐2@Ch]_cry_‐CA groups did not differ significantly from the untreated group, suggesting that cryogel formulation and CA loading play a critical role in enhancing systemic protein content. Total hemolymph protein increase may reflect an elevated synthesis of immune‐related proteins (e.g., antimicrobial peptides or stress proteins), though specific protein profiles were not assessed in this study. [Ch‐3]_cry_‐CA demonstrated the most effective performance in terms of hemocyte proliferation, wound healing, and melanization, while [Ch‐2@Ch]_cry_‐CA was superior in antioxidant defense, as evidenced by its significantly higher SOD activity and lower MDA levels. Although all cryogels contributed to a reduction in MDA levels compared to the untreated group, [Ch‐2@Ch]_cry_‐CA exhibited a significantly lower MDA concentration than all other cryogels, indicating its superior efficacy in mitigating lipid peroxidation. According to these findings, Ch cryogels could alone enhance the immune system; however, they could further increase immunological and antioxidant defenses when they were loaded with CA. Future research should focus on the interaction between CA and Ch cryogels to elucidate their impact on immune response mechanisms, enhancing immune cell function at the molecular level. To fully contextualize the scope and applicability of these findings, it is also important to consider the limitations of the experimental model used in this study. While *G. mellonella* offers a practical and ethically acceptable in vivo model for assessing innate immune responses and wound healing, its lack of an adaptive immune system and physiological divergence from mammals limit the direct translatability of results. Consequently, complementary studies in vertebrate models are warranted to confirm the biomedical relevance of the findings. The current study concentrated on the structural and physicochemical characteristics of the cryogels; however, we appreciate that thorough biocompatibility evaluations, including cytocompatibility and haemolysis testing, are crucial for biomedical application and will be explored in subsequent research. Furthermore, the present research revealed that the interactions of Ch cryogels and CA‐loaded forms with *G. mellonella* for the first time in order to guide other researchers who will work in this field.

## Author Contributions

**Sema Ekici:** conceptualization, methodology, investigation, realization of cryogel synthesis and characterization, visualization, data curation, supervision, writing – reviewing and editing. **Serhat Kaya:** conceptualization, methodology, investigation, experimental (with *G. mellonella*), visualization, data curation, writing – reviewing and editing. **Gürkan Durucu:** realization of cryogel synthesis and characterization, investigation.

## Conflicts of Interest

The authors declare no conflicts of interest.

## Supporting information


**Data S1:** bip70042‐sup‐0001‐Figure.docx.

## Data Availability

The data that support the findings of this study are available from the corresponding author upon reasonable request.
