# Supplementary material for: In Vivo Wound Healing and Immune Response Studies of Chitosan Cryogels With Invertebrate Model Organism Galleria mellonella
Source: Biopolymers. 2025 Jul 30;116(5):e70042. doi: 10.1002/bip.70042 (PMC12309346; doi:10.1002/bip.70042)
Supplement: Supplementary file 1 — Data S1: bip70042‐sup‐0001‐Figure.docx. [file BIP-116-e70042-s001.docx]

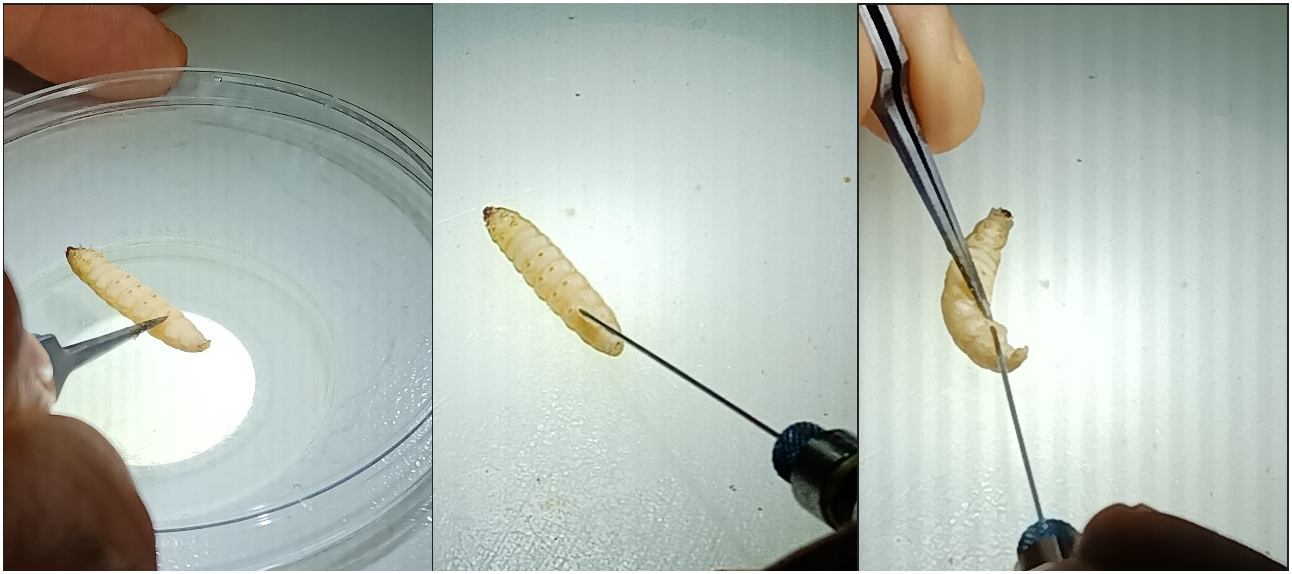


S1. Insertion of cryogels into the body of larvae via hamilton syringe through the last of their prolegs.


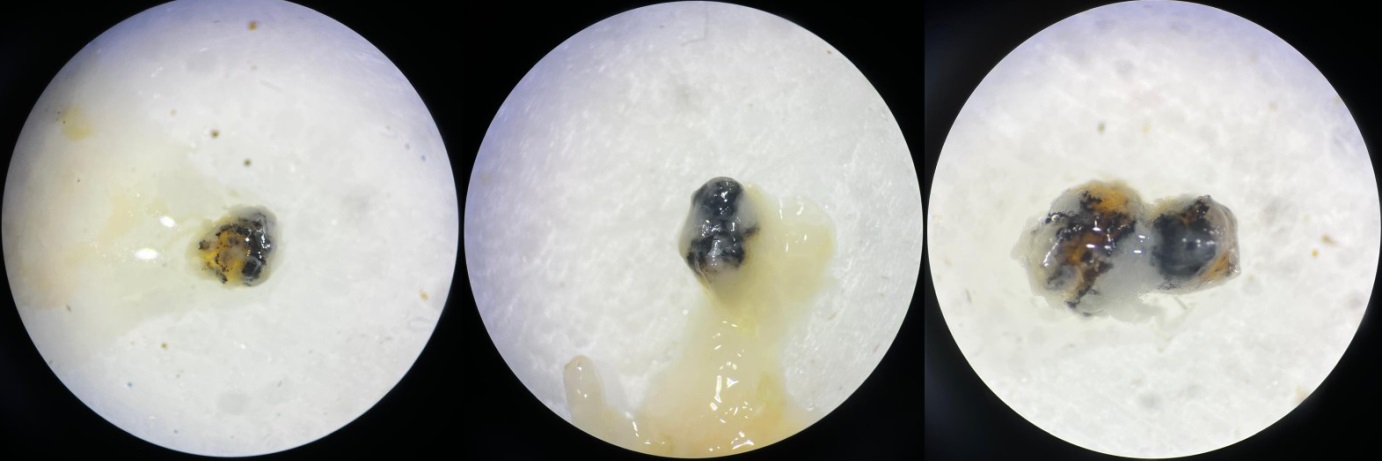


S2. The photos showing melanization on cryogels randomly selected.


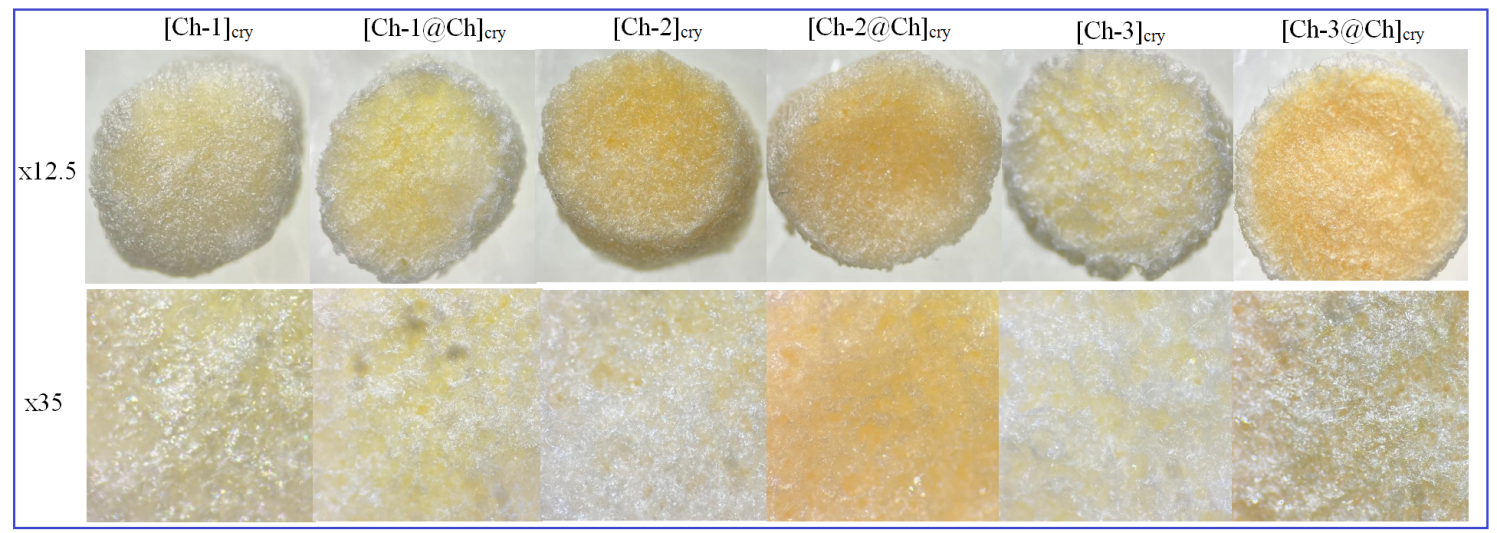


S3. The stereomicroscope images of cryogels with different magnification.


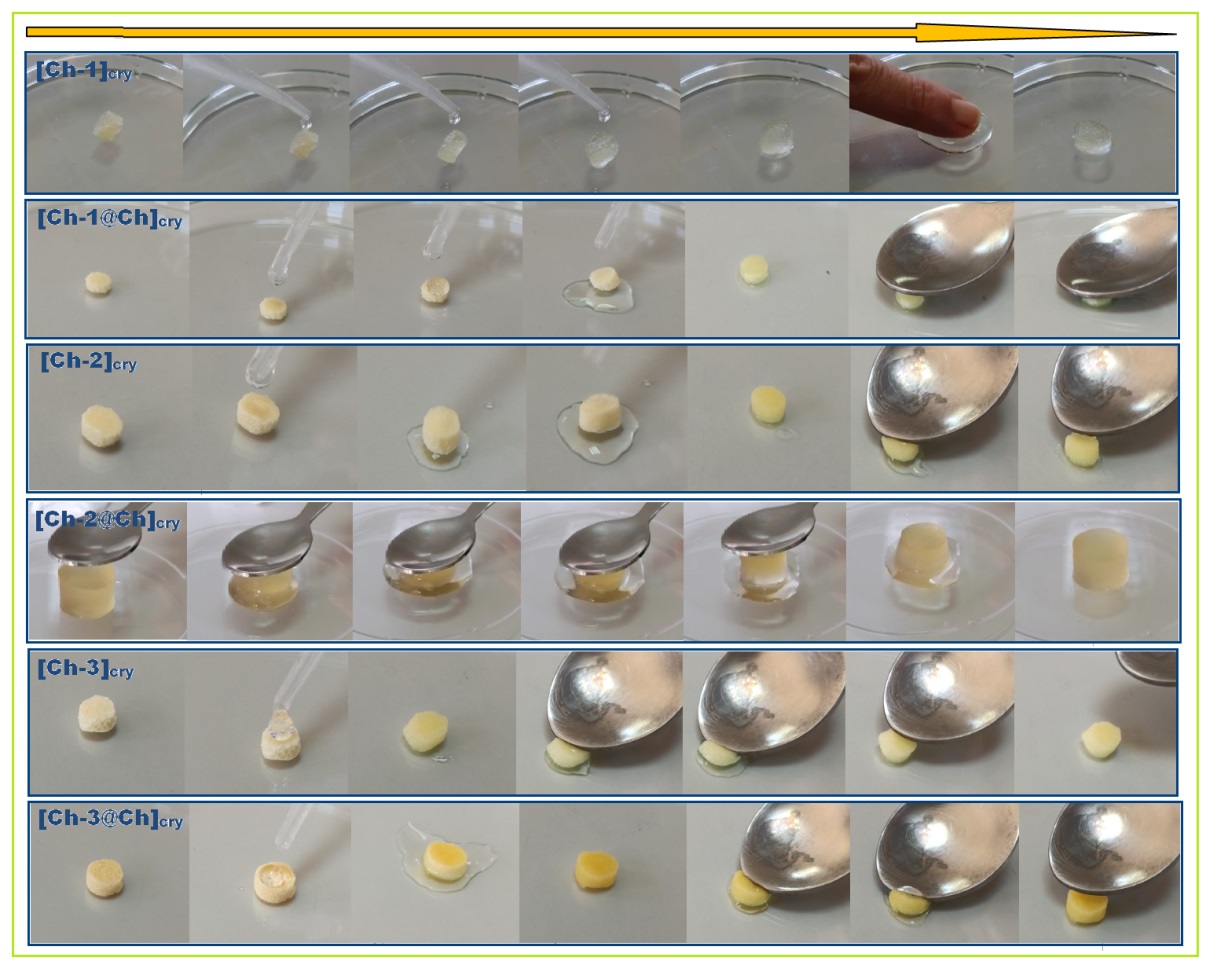


S4. Photographs of the swelling and the compressive-shape recovery (left to right) characteristics of cryogels.


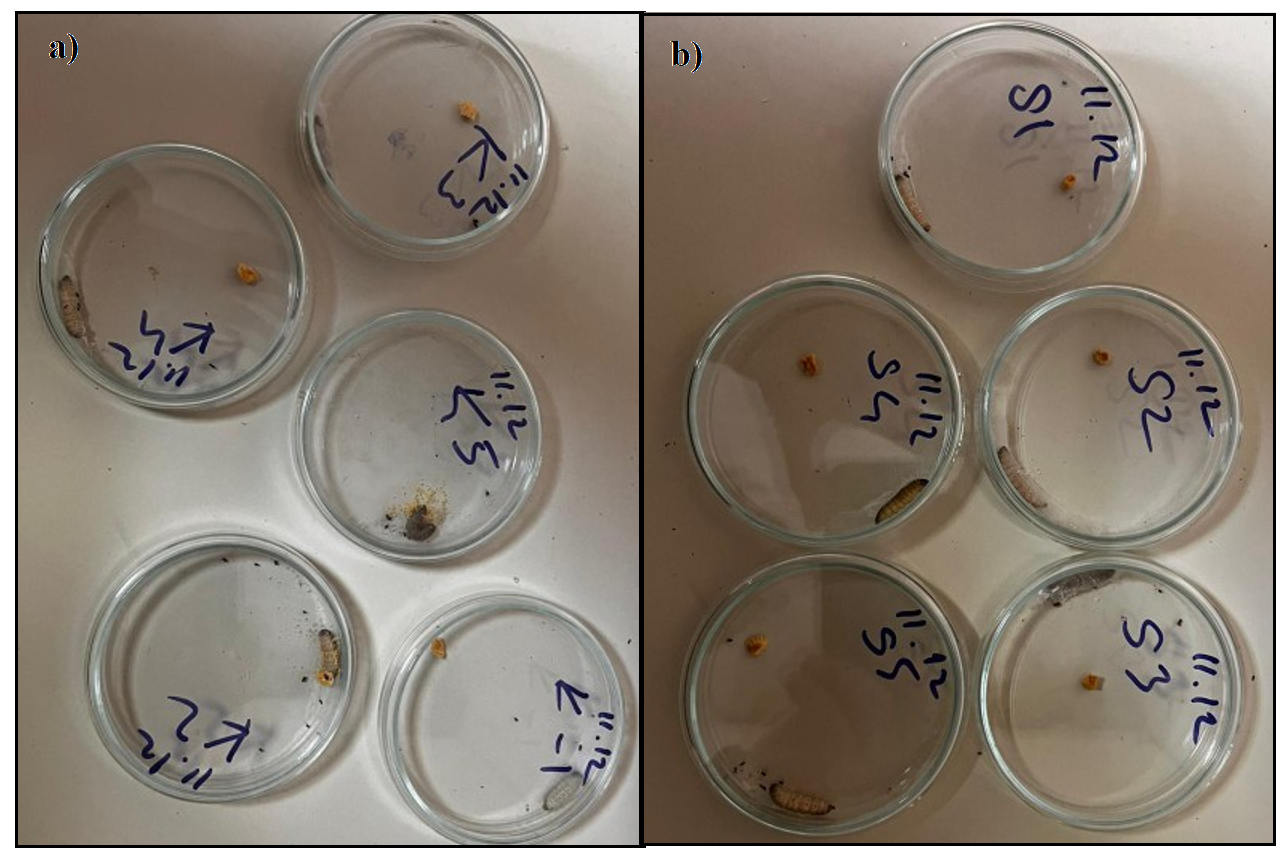


S5. Application of the feeding test to the cryogels, a) [Ch-3]_cry_ and [Ch-2@Ch]_cry_, b) [Ch-3]_cry_-CA and [Ch-2@Ch]_cry_-CA. *G. mellonella* larvae are randomly placed in the petri dishes.
